# Supplementary material for: Rates and causes of mortality among children and young people with and without intellectual disabilities in Scotland: a record linkage cohort study of 796 190 school children
Source: BMJ Open. 2020 Aug 9;10(8):e034077. doi: 10.1136/bmjopen-2019-034077 (PMC7418667; doi:10.1136/bmjopen-2019-034077)
Supplement: Supplementary data [file bmjopen-2019-034077supp001.pdf]

Supplementary – Appendix 1 - Reported standardised mortality ratios for children and young people with intellectual disabilities

Author	Country	SMR overall (95% confidence interval) at all ages	Number of deaths of children/young people	SMR for children/young people
McGuigan et al (1995)[1]	England	-	18 aged 0-19 y	1.7 (0.3, 4.9) for males aged 0-9 y*<br>3.1 (0.6, 9.0) for females aged 0-9 y*<br>17.1 (2.1, 61.8) for males aged 2-14 y*<br>20.7 (2.5, 74.7) for females aged 2-14 y*<br>22.2 (6.1, 56.8) for males aged 7-19 y*<br>34.0 (9.3, 87.1) for females aged 7-19 y*
Forsgren et al (1996)[2]	Sweden	2.0 (1.7, 2.3)	12 aged 0-19 y	15.5 (10.2, 23.6) aged 0-19 y
Patja et al (2001)[3]	Finland	-	50 aged 2-19 y; 29 profound ID, 5 Severe ID, 7 moderate ID, 8 mild ID	Death from disease: 2.6 for males, 1.7 for females aged 2-19 y<br>Accidental death: 0.1 for males, 0.1 for females aged 2 -19 y
Decouflé & Autry (2002)[4]	USA	_	23 aged 10-20 y	3.3 (2.1, 5.0) aged 10-19 y<br>1.4 (0.6, 2.9) with mild ID aged 10-19 y<br>8.4 (4.8, 13.6) with severe ID aged 10-19 y
Shavelle et al. (2014) [5]	USA	1.65 for mild and moderate ID<br>1.85 for severe and profound ID	59 aged 5-19 y mild and moderate ID<br>7 aged 5-19 y severe and profound ID	1.26** for mild and moderate ID aged 5-19 y<br>8.30** for severe and profound ID aged 5-19 y
Ouellette-Kuntz et al (2015)[6]	Canada	2.5 (2.1, 2.9)	11 males aged 0-19 y<br>3 females aged 0-19 y	21.6 (10.8, 38.7) for males aged 0-19 y<br>18.1 (3.7, 53.0) for females aged 0-19 y
Arvio et al (2016)[7]	Finland	2.3 (2.2, 2.4) for mild ID; 2.0 (1.9, 2.1) for males, 2.8 (2.6, 3.0) for females<br>3.4 (3.3, 3.5) for severe ID; 2.6 (2.5, 2.7) for males, 5.2 (5.0, 5.5) for females	8 with mild ID aged 0-14 y;<br>4 males, 4 females<br>110 with severe ID aged 0-14 y; 42 males, 68 female	4.2 (1.8, 8.3) with mild ID aged 0-14 y;<br>3.2 (0.9, 8.1) for males, 6.3 (1.7, 16.2) for females<br>13.3 (10.9, -) with severe ID aged 0-14 y;<br>8.2 (5.9, 11.1) for males, 21.4 (16.6, 27.1) for females
McCarron et al (2015)[8]	Republic of Ireland	3.9 (3.7, 4.0)	272 aged 0-19 y	6.9 (5.9, 7.5) aged 0-19 y
Lauer et al (2015) [9]	USA	1.19	58 age 18-24 y	5.9** aged 18-24 y
Glover et al (2017)[10]	England	3.2 (2.9, 3.4)	33 aged 0-17 y	30.4 (18.3, 47.5) aged 0-9 y<br>17.3 (9.4, 29.0) aged 10-17 y
Trollor et al (2017) [11]	Australia	1.3 (1.2-1.5)	aged 20-24 y not provided	4.5** aged 20-24 y**
Bourke et al (2017)[12]	Australia	-	326 aged 1-27 y	aHR=6.0 (4.8, 7.6) aged 1-5 y<br>aHR=12.6 (9.0, 17.7) aged 6-10 y<br>aHR=4.9 (3.9, 6.1) aged 11-25 y

aHR=adjusted hazard ratio; ID=intellectual disabilities; SMR=standardised mortality ratio; y=years; *by individual birth cohorts **mortality ratio calculated from age-specific mortality rates

Supplementary References

1. McGuigan SM, Hollins S, Attard M. Age-specific standardized mortality rates in people with learning disability. *J Intellect Disabil Res* 1995;39(6):527-531
2. Forsgren L, Edvinsson SO, Nystrom L, et al. Influence of epilepsy on mortality in mental retardation: An epidemiologic study. *Epilepsia* 1996;37(10):956–963.
3. Patja K, Mölsä P, Iivanainen M. Cause-specific mortality of people with intellectual disability in a population-based, 35-year follow-up study. *J Intellect Disabil Res* 2001;45:30–40
4. Decouflé P, Autry A. Increased mortality in children and adolescents with developmental disabilities. *Paediatr Perinat Epidemiol* 2002;16(4):375–382
5. Shavelle RM, Sweeney LH, Brooks JC. Comparative Mortality of Persons With Intellectual Disability in California: An Update (2000-2010) *J Insur Med* 2014; 44(3):158-63
6. Ouellette-Kuntz H, Shooshtari S, Balogh R, et al. Understanding information about mortality among people with intellectual and developmental disabilities in Canada. *J Appl Res Intellect Disabil* 2015;28(5):423–435
7. Arvio M, Salokivi T, Tiitinen A, et al. Mortality in individuals with intellectual disabilities in Finland. *Brain Behav* 2016;6(2):1–4.
8. McCarron M, Carroll R, Kelly C, et al. Mortality rates in the general Irish population compared to those with an intellectual disability from 2003 to 2012. *J Appl Res Intellect Disabil* 2015;28(5):406–413
9. Lauer E, McCallion P. Mortality of People with Intellectual and Developmental Disabilities from Select US State Disability Service Systems and Medical Claims Data *J Appl Res Intellect Disabil* 2015;28;394-405
10. Glover G, Williams R, Heslop P, et al. Mortality in people with intellectual disabilities in England. *J Intellect Disabil Res* 2017;61(1):62–74
11. Trollor J, Srasuebkul P, Xu H, et al. Cause of death and potentially avoidable deaths in Australian adults with intellectual disability using retrospective linked data. *BMJ Open* 2017;7:e013489.
12. Bourke J, Nembhard WN, Wong K, et al. Twenty-Five Year Survival of Children with Intellectual Disability in Western Australia. *J Pediatr* 2017;188:232-239.e2.

Supplementary – Appendix 2. Kaplan-Meier survival estimates for pupils with and without intellectual disabilities
