# Supplementary figures and images for: Rates and causes of mortality among children and young people with and without intellectual disabilities in Scotland: a record linkage cohort study of 796 190 school children
Source: BMJ Open. 2020 Aug 9;10(8):e034077. doi: 10.1136/bmjopen-2019-034077 (PMC7418667; doi:10.1136/bmjopen-2019-034077)

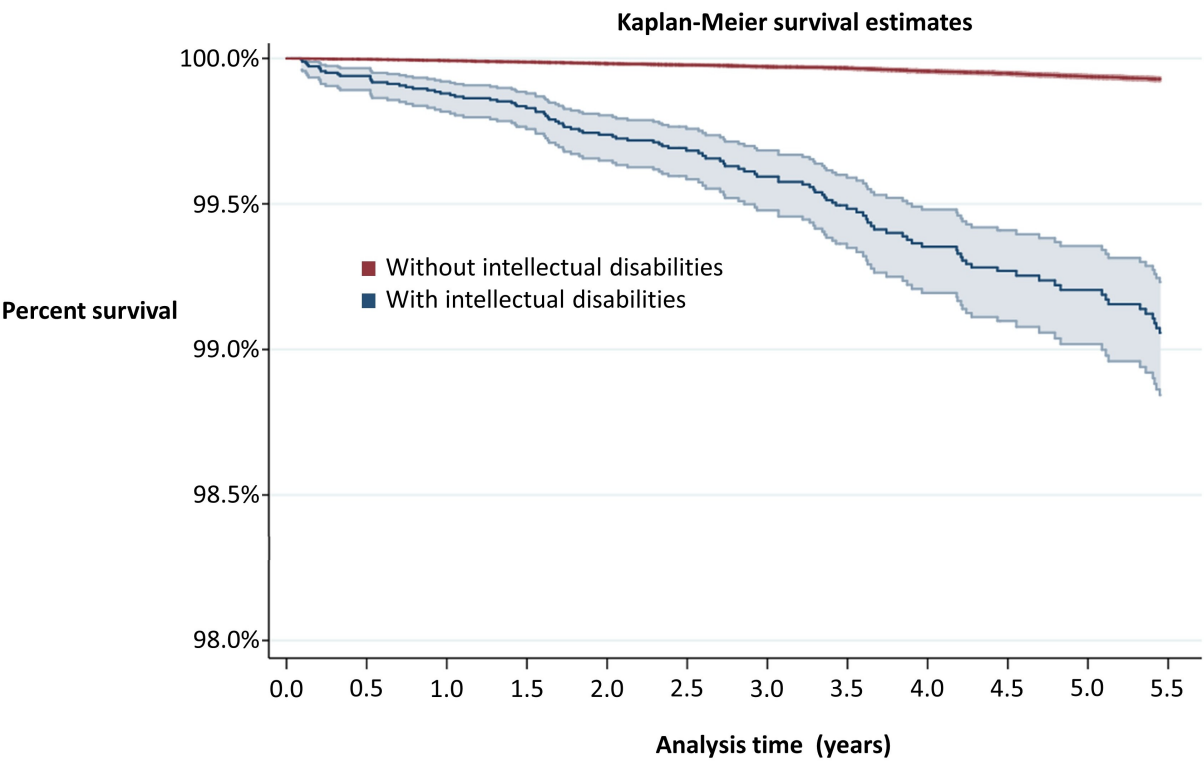

Supplement: Supplementary data [file bmjopen-2019-034077supp002.pdf]
