# Supplementary material for: Rates and causes of mortality among children and young people with and without intellectual disabilities in Scotland: a record linkage cohort study of 796 190 school children
Source: BMJ Open. 2020 Aug 9;10(8):e034077. doi: 10.1136/bmjopen-2019-034077 (PMC7418667; doi:10.1136/bmjopen-2019-034077)
Supplement: Author's manuscript [file bmjopen-2019-034077.draft_revisions.pdf]

BMJ Open

BMJ Open is committed to open peer review. As part of this commitment we make the peer review history of every article we publish publicly available.

When an article is published we post the peer reviewers' comments and the authors' responses online. We also post the versions of the paper that were used during peer review. These are the versions that the peer review comments apply to.

The versions of the paper that follow are the versions that were submitted during the peer review process. They are not the versions of record or the final published versions. They should not be cited or distributed as the published version of this manuscript.

BMJ Open is an open access journal and the full, final, typeset and author-corrected version of record of the manuscript is available on our site with no access controls, subscription charges or pay-per-view fees (http://bmjopen.bmj.com).

If you have any questions on BMJ Open's open peer review process please email info.bmjopen@bmj.com

BMJ Open

Rates and causes of mortality among children and young people with intellectual disabilities in Scotland: a record linkage cohort study of 800,457 schoolchildren

Journal:	*BMJ Open*
Manuscript ID	bmjopen-2019-034077
Article Type:	Original research
Date Submitted by the Author:	04-Sep-2019
Complete List of Authors:	Smith, Gillian; University of Glasgow Institute of Health and Wellbeing<br>Fleming, Michael ; University of Glasgow, Institute of Health and Wellbeing<br>Kinnear, Deborah; University of Glasgow, Institute of Health and Wellbeing<br>Henderson, Angela; University of Glasgow, Institute of Health and Wellbeing<br>Pell, J. P.; University of Glasgow, Institute of Health and Wellbeing<br>Melville, Craig; University of Glasgow, Institute of Health and Wellbeing<br>Cooper, Sally-Ann; University of Glasgow, Institute of Health and Wellbeing
Keywords:	intellectual disabilities, mortality, death, children, young people

SCHOLARONE™
Manuscripts

I, the Submitting Author has the right to grant and does grant on behalf of all authors of the Work (as defined in the below author licence), an exclusive licence and/or a non-exclusive licence for contributions from authors who are: i) UK Crown employees; ii) where BMJ has agreed a CC-BY licence shall apply, and/or iii) in accordance with the terms applicable for US Federal Government officers or employees acting as part of their official duties; on a worldwide, perpetual, irrevocable, royalty-free basis to BMJ Publishing Group Ltd ("BMJ") its licensees and where the relevant Journal is co-owned by BMJ to the co-owners of the Journal, to publish the Work in this journal and any other BMJ products and to exploit all rights, as set out in our licence.

The Submitting Author accepts and understands that any supply made under these terms is made by BMJ to the Submitting Author unless you are acting as an employee on behalf of your employer or a postgraduate student of an affiliated institution which is paying any applicable article publishing charge ("APC") for Open Access articles. Where the Submitting Author wishes to make the Work available on an Open Access basis (and intends to pay the relevant APC), the terms of reuse of such Open Access shall be governed by a Creative Commons licence – details of these licences and which Creative Commons licence will apply to this Work are set out in our licence referred to above.

Other than as permitted in any relevant BMJ Author's Self Archiving Policies, I confirm this Work has not been accepted for publication elsewhere, is not being considered for publication elsewhere and does not duplicate material already published. I confirm all authors consent to publication of this Work and authorise the granting of this licence.

For peer review only

Rates and causes of mortality among children and young people with intellectual disabilities in Scotland: a record linkage cohort study of 800,457 schoolchildren

Smith, G.S., Fleming, M., Kinnear, D., Henderson, A., Pell, J.P., Melville, C., Cooper, S-A.

Corresponding author: Professor Sally-Ann Cooper; Sally-Ann.Cooper@glasgow.ac.uk

For peer review only

Abstract

Objectives: To investigate mortality rates and causes in children and young people with intellectual disabilities.

Design: Retrospective cohort, with individual record-linkage between Scotland's annual pupil census and National Records of Scotland death register.

Setting: General community.

Participants: Pupils receiving Local Authority funded schooling in Scotland, 2008-2013, with an Additional Support Need due to intellectual disabilities, compared with other pupils.

Main outcome measures: Deaths up to 2015: age of death, age and sex-standardised mortality ratios (SMRs) and causes of death including cause and sex-specific age-SMRs.

Results: 18,595/1,016,868 (1.8%) pupils had intellectual disabilities. 106 died over 68,539 person-years (crude mortality rate=154/100,000 person-years), compared with 461 controls over 3,692,459 person-years (crude mortality rate=13/100,000 person-years). Age-SMR was 12.1 (95% CI 10.0, 14.6); 16.4 (12.0, 22.3) for female pupils, 9.6 (7.5, 12.2) for male pupils. Most common main underlying causes were diseases of the nervous system, then congenital anomalies; most common all-contributing causes were diseases of the nervous system, then respiratory system; most common specific contributing causes were cerebral palsy, pneumonia, respiratory failure, and epilepsy. For all contributing causes, SMR was 97.6 (69.0, 138.0) for congenital anomalies, 71.7 (55.2, 93.2) for nervous system, 62.9 (36.5, 108.4) for digestive system, 53.6 (41.2, 69.8) for respiratory system, 30.0 (16.6, 54.1) for endocrine, 14.0 (8.5, 23.0) for circulatory system. External causes accounted for 46% of

For peer review only - http://bmjopen.bmj.com/site/about/guidelines.xhtml

control deaths, but SMR was still higher (3.5 {2.2, 5.8}) for pupils with intellectual disabilities. Deaths amenable to good care were common.

Conclusions: Pupils with intellectual disabilities were much more likely to die than their peers, and had a different pattern of causes, including amenable deaths across a wide range of disease categories. Targeted improvements in care should be developed and delivered to reduce this inequality. Clinicians, carers, educators, and policy makers should be aware of mortality risks in order to make improvements.

Strengths and limitations of this study

- Novel use of education records and record linkage to death records to study mortality in an unselected cohort of children and young people with intellectual disabilities
- Due to the use of a whole country population these results are well-powered and generalisable
- Despite comprising a whole country population, our study was not large enough to delineate cause-specific mortality ratios by gender
- This study was limited by lack of information on the severity or cause of intellectual disabilities
- Reliance on death certificate data is limited by inconsistencies in reporting of cause of death

Key words

Intellectual disabilities, mortality, death, children, young people

For peer review only - http://bmjopen.bmj.com/site/about/guidelines.xhtml

Introduction

Children and young people with intellectual disabilities have a much higher prevalence of physical and mental ill-health compared to the general population[1-3]. The life expectancy of people with intellectual disabilities has been reported to be about 20 years shorter than in the general population, or 28 years shorter specifically for people with Down syndrome[4-7]. While the actual number of deaths in childhood is smaller than in adults, mortality studies comparing people with intellectual disabilities with the general population have shown increased risk ratios in younger age groups compared to adults. However, the excess reported risk varies considerably between studies, and not all studies are comparable due to e.g. reporting deaths within different age ranges, and additionally some have small sample sizes and wide confidence intervals. Reported standardised mortality ratios (SMR) have ranged from 3.3 (95% confidence interval [CI] 2.1, 5.0) in young people aged 10-19 years[8], to 17.3 (95% CI 9.4, 29.0) in young people aged 10-17 years[9]; from 2.6 in males aged 2-19 years and 1.7 in females aged 2-19 years[10], to 21.6 (95% CI 10.8, 38.7) in males aged 0-19 years and 18.1 (95% CI 3.7, 53.0) in females aged 0-19 years[11], and have been reported to be 30.4 (95% CI 18.4, 47.5) in children aged 0-9 years[9]. Table 1 summarises previously reported SMRs in children and young people with intellectual disabilities.

For peer review only

For peer review only - http://bmjopen.bmj.com/site/about/guidelines.xhtml

Table 1. Reported standardised mortality ratios for children and young people with intellectual disabilities

Author	Country	SMR overall (95% confidence interval) at all ages	Number of deaths of children/young people	SMR for children/young people
McGuigan et al (1995)[12]	England	-	18 aged 0-19 y	1.7 (0.3, 4.9) for males aged 0-9 y*<br>3.1 (0.6, 9.0) for females aged 0-9 y*<br>17.1 (2.1, 61.8) for males aged 2-14 y*<br>20.7 (2.5, 74.7) for females aged 2-14 y*<br>22.2 (6.1, 56.8) for males aged 7-19 y*<br>34.0 (9.3, 87.1) for females aged 7-19 y*
Forsgren et al (1996)[13]	Sweden	2.0 (1.7, 2.3)	12 aged 0-19 y	15.5 (10.2, 23.6) aged 0-19 y
Decouflé & Autry (2002)[8]	USA	_	23 aged 10-20 y	3.3 (2.1, 5.0) aged 10-19 y<br>1.4 (0.6, 2.9) with mild ID aged 10-19 y<br>8.4 (4.8, 13.6) with severe ID aged 10-19 y
Patja et al (2001)[10]	Finland	-	50 aged 2-19 y; 29 profound ID, 5 Severe ID, 7 moderate ID, 8 mild ID	Death from disease: 2.6 for males, 1.7 for females aged 2-19 y<br>Accidental death: 0.1 for males, 0.1 for females aged 2 -19 y
Ouellette-Kuntz et al (2015)[11]	Canada	2.5 (2.1, 2.9)	11 males aged 0-19 y<br>3 females aged 0-19 y	21.6 (10.8, 38.7) for males aged 0-19 y<br>18.1 (3.7, 53.0) for females aged 0-19 y
Arvio et al (2016)[14]	Finland	2.3 (2.2, 2.4) for mild ID; 2.0 (1.9, 2.1) for males, 2.8 (2.6, 3.0) for females<br>3.4 (3.3, 3.5) for severe ID; 2.6 (2.5, 2.7) for males, 5.2 (5.0, 5.5) for females	8 with mild ID aged 0-14 y;<br>4 males, 4 females<br>110 with severe ID aged 0-14 y; 42 males, 68 female	4.2 (1.8, 8.3) with mild ID aged 0-14 y;<br>3.2 (0.9, 8.1) for males, 6.3 (1.7, 16.2) for females<br>13.3 (10.9, -) with severe ID aged 0-14 y;<br>8.2 (5.9, 11.1) for males, 21.4 (16.6, 27.1) for females
McCarron et al (2015)[15]	Republic of Ireland	3.9 (3.7, 4.0)	272 aged 0-19 y	6.9 (5.9, 7.5) aged 0-19 y
Glover et al (2017)[9]	England	3.2 (2.9, 3.4)	33 aged 0-17 y	30.4 (18.3, 47.5) aged 0-9 y<br>17.3 (9.4, 29.0) aged 10-17 y
Bourke et al (2017)[16]	Australia	-	326 aged 1-27 y	aHR=6.0 (4.8, 7.6) aged 1-5 y<br>aHR=12.6 (9.0, 17.7) aged 6-10 y<br>aHR=4.9 (3.9, 6.1) aged 11-25 y

aHR=adjusted hazard ratio; ID=intellectual disabilities; SMR=standardised mortality ratio; y=years; *by individual birth cohorts

For peer review only - http://bmjopen.bmj.com/site/about/guidelines.xhtml

Most of these studies do not report causes of death among children and young people with intellectual disabilities. Bourke et al. (2017)[16] reported the most common causes of death in children, young people, and adults with intellectual disabilities aged 1-25 years to be respiratory infection (34%), with an additional 10% having an aspiration related cause, congenital heart defects (15%), and accidents (11%). Compared to children and young people who did not have intellectual disabilities, their causes of death by ICD 10 chapter were more likely to be attributed to the nervous system, endocrine, nutritional and metabolic diseases, or congenital malformations, and less likely to be attributed to conditions originating in the perinatal period, external causes, or injury or poisoning[16]. However, a small proportion of these deaths were among adults rather than children and young people, who may have a different cause of death profile. Patja et al. (2001)[10] reported respiratory diseases to be the most common underlying/immediate cause of death in children and young people with intellectual disabilities aged 2-19 years, with a relative risk of 5.8 (95% CI 4.4, 15.6) in males and 4.3 (0.3, 4.7) in females, and did not find any other causes (infectious diseases, tumours, vascular diseases, diseases of digestive system, accidents and poisonings, or other causes) to differ from those expected in the general population. However, the study was limited by small sample size. Durvasula et al. (2009)[17] reported 7 of 14 deaths among young people with intellectual disabilities aged 10-24 years were attributed to the respiratory system (pneumonia, aspiration).

Adults with intellectual disabilities are over-represented in deaths which would have been amenable to treatment by timely and effective health care[4-5, 9]. However, there is limited evidence on whether children and young people with intellectual disabilities also experience such amenable deaths more commonly than other children and young people, as most authors who have reported cause-specific mortality do so grouped across all ages, due to sample sizes.

Overall, studies on mortality in children with intellectual disabilities are few in number, mostly small in size, and results are variable, and they do not report on causes of death. Hence, the aim of this longitudinal cohort study is to compare all-cause and cause-specific mortality in Scotland's school-aged population with and without intellectual disabilities.

Methods

We used education data from Scotland's annual pupil census between 2008 and 2013, to establish a cohort of children and young people with and without intellectual disabilities. We used individual record linkage to the National Records for Scotland (NRS) deaths registry, to ascertain all deaths up to February 2015 in Scotland.

For peer review only - http://bmjopen.bmj.com/site/about/guidelines.xhtml

The Scottish annual pupil census is completed in September each year and provides information on all children attending Local Authority funded primary, secondary, and special schools in Scotland, or funded placements in alternative schools, which includes 95% of the entire population of children and young people in Scotland. This information includes whether the child has a record of Additional Support Needs, and the type of Additional Support Need. It is held by the Scottish exchange of education data (ScotXed).

We excluded non-singleton births (available for Scottish-born pupils only, identified from linkage to maternity records). We included all pupils with records of Additional Support Need due to intellectual disabilities between 2008 and 2013, between the ages 4 and 19 years old. Only pupils with intellectual disabilities recorded in at least two different school years were included in the intellectual disabilities group. Pupils who were included in at least two pupil censuses over the study period and had no record of intellectual disabilities or autism were used as the comparison group. Pupils with autism were also excluded from controls, due to high comorbidity with intellectual disabilities.

The pupil census also includes data on age, gender, ethnicity and Scottish Index of Multiple Deprivation 2012 (SIMD)[18]. Derived from individual pupil postcode of residence, SIMD is a composite of seven indices to indicate the extent of neighbourhood deprivation. SIMD was divided into quintiles according to the general population. Data on disability requirements including physical (e.g. visual, hearing, or physical impairments), communication, or curriculum needs are also listed.

Explorative statistical analysis using t-tests and χ^2-tests were employed to investigate characteristics of pupils with intellectual disabilities compared to their peers in the comparison group. Differences in age of death were explored using t-tests. Crude mortality rates were calculated. Since only those pupils who attended school in at least two of the years over our observed study period were eligible, the entry to the study was defined as the date of their second pupil census record to account for immortal time bias in the first year. The mortality rates were indirectly standardised for both males and females using the expected age-specific mortality rates derived from the comparison group to calculate age-standardised mortality ratios (SMRs). SMRs were subsequently stratified by age, into childhood (aged 5-14 years) and young people (aged ≥15 years), and by sex. The SMRs were also re-calculated to exclude deaths from external causes. This was to investigate whether the over-representation of female deaths in people with intellectual disabilities compared to the general population[14-16] is related to the large proportion of male deaths from external causes in the general population[19].

For cause of death analyses, the underlying cause of death is defined internationally[20] as the disease or injury which initiated the chain of morbid events leading directly to death, or the accident/act which

For peer review only - http://bmjopen.bmj.com/site/about/guidelines.xhtml

produced the fatal injury. We also used a broader definition to analyse all-contributing causes, that included all deaths with any mention on the death certificate related to the cause, combining both the underlying cause, with secondary, or contributing factors. While the same ICD 10 codes are used, it is important to note that one death may have several other, additional causes as contributing factors, all of which are counted in figures reporting “all-contributing causes”.

For the underlying causes of death, the total number of deaths in each ICD 10 chapter were collated, and this was then repeated for specific causes listed within chapters. Any errors or ambiguous deaths were listed as an unknown cause. All deaths where the underlying cause was ill-defined, as defined in ICD 10[20], listed as ICD10 codes “R00-R94” “R95-R99”, were also re-classified as “unknown”. Next, the breakdown of all-contributing causes were analysed, by collating number of deaths in each ICD 10 chapter. Indirect age-standardisation of cause-specific mortality ratios was carried out using 5-year age-bands, except for categories that had fewer than ten deaths.

The Office of National Statistics (ONS) revised definition of avoidable mortality for children and young people[21] defined avoidable mortality as either amenable mortality (avoidable through good quality healthcare even after a condition has developed) or preventable mortality (avoidable through incidence reduction via public health interventions) or both. This list of ICD 10 causes was used to determine the occurrence of avoidable deaths. The rates and age-standardised mortality ratios (age-standardised using 5-year age-bands) for avoidable, amenable, and preventable mortality were calculated, except where there were for fewer than ten deaths per chapter. In keeping with the ONS avoidable mortality methodology[22], avoidable mortality rates based on fewer than twenty deaths were labelled as unreliable.

All statistical analyses were undertaken using Stata, version 15.0 (StataCorp).

Personal and Patient involvement

This study was undertaken in the Scottish Learning Disabilities Observatory due to the growing concern among people with intellectual disabilities and their families around mortality. Its steering group includes people with intellectual disabilities, and partners from third sector organisations. Results from this study will be disseminated to people with intellectual disabilities and their families in an easy-read version via the Scottish Learning Disabilities Observatory website and newsletters.

Results

Out of 1,016,868 pupils in the census between 2008 and 2013, 18,595 (1.8%) were identified as having Additional Support Needs due to intellectual disabilities in at least two school census years. There

For peer review only - http://bmjopen.bmj.com/site/about/guidelines.xhtml

For peer review only

were 976,089 pupils without any records of intellectual disabilities or autism. Of these, 781,862 pupils attended school for at least two years over the study period and were designated as controls.

Using data from the pupils' first year in the Census, pupils with intellectual disabilities were more likely to be male, and more likely to reside in areas of greater neighbourhood deprivation, and to have been registered for free school meals, compared to their peers (Table 2). Pupils with intellectual disabilities were also more likely to require adaptations in school, including physical adaptations, communication and curriculum adaptations. The majority of the study population were identified as having white (white -Scottish, -British or -other) ethnicity.

For peer review only

For peer review only - http://bmjopen.bmj.com/site/about/guidelines.xhtml

Table 2. Demographic information for pupils with intellectual disabilities compared with their peers

Demographic information	Intellectual disabilities pupils		Control pupils	
**Total, n**	18,595		781,862	
**Male sex, n (%)**	12,107	(65%)**	391,367	(50%)
**Free school meals, n (%)**	9,677	(52%)**	198,038	(25%)
**Disability adaptations, n (%)**				
Physical adaptation	2010	(11%)**	1863	(0.2%)
Curriculum adaptation	6,745	(36%)**	6,459	(0.8%)
Communication adaptation	3,623	(19%)**	1,804	(0.2%)
**SIMD quintile, n (%)**				
1 (most deprived)	6007	(32%)	172,654	(22%)
2	3946	(21%)	150,985	(19%)
3	3415	(18%)	151,958	(19%)
4	2907	(16%)	157,287	(20%)
5 (least deprived)	2320	(13%)**	148,978	(19%)
**Ethnicity, n (%)**				
White [a]	17,347	(93%)	726,824	(93%)
Asian [a]	523	(3%)	23,726	(3%)
Mixed or multiple ethnicities	265	(1%)	11,585	(2%)
African, Caribbean or black	100	(<1%)	4971	(<1%)
Other ethnic groups	132	(<1%)	6602	(<1%)
Not disclosed / or unknown	228	(1%)	8154	(1%)

** $p \leq 0.001$, χ^2 compared to control group (For SIMD - χ^2 test was across all categories, overall p value)

[a] (white -Scottish, -British, -Other) (Asian -Indian/British/Scottish, -Pakistani/British/Scottish, -Bangladeshi/British/Scottish, -Chinese/British/Scottish

For peer review only

For peer review only - http://bmjopen.bmj.com/site/about/guidelines.xhtml

Mortality analysis

Linking the pupil census population to the NRS registry of deaths up to February 2015 resulted in the equivalent of 3,760,998 person-years of follow up. There were 567 deaths identified in the study population during this period. There were 106 deaths (0.6%) among children and young people with intellectual disabilities over 68,539 person-years, which translated to a crude mortality rate of 154 deaths per 100,000 person-years (95% CI 128, 188). In the control group, there were 461 deaths (<0.1%) over 3,692,459 person-years, which translated to a crude mortality rate of 13 deaths per 100,000 person-years (95% CI 12, 14). The mean age of death among children and young people with intellectual disabilities was 14.3 years (95% CI 13.3 to 15.1) which was significantly lower (p<0.001) than controls where the mean age of death was 16.1 years (15.7 to 16.5). Sixty-two percent of deaths among children with intellectual disabilities occurred in males which was equivalent to the sex distribution in the whole intellectual disabilities cohort (p=0.538). Among controls, 61% of deaths occurred in males in spite of them accounting for only 50% of this group (p<0.001). Over 50% of deaths among pupils with intellectual disabilities occurred during childhood (<15 years old), compared to 29% of deaths among controls.

The all-cause SMR was 12.1 (95% CI 10.0, 14.6), as shown in Figure 1. The SMR was higher for female pupils than male pupils with intellectual disabilities; female SMR 16.4 (12.0, 22.3) versus male SMR 9.6 (7.5, 12.2). Exclusion of external causes of death resulted in a considerable increase in the all-cause SMR for both females and males with intellectual disabilities; overall SMR was 21.4 (17.6, 26.0), female SMR 25.2 (18.5, 34.3) versus male SMR 19.0 (14.8, 24.5).

The childhood (aged 5-14 years) SMR was 22.5 (17.3, 29.3) and was higher for females than males with intellectual disabilities; female SMR 30.0 (19.5, 46.0) versus male SMR 18.1 (13.0, 25.4). For young people (≥ 15 years old) SMR was 8.1 (6.1, 10.6) and was also higher for females than males with intellectual disabilities; female SMR 10.9 (7.0, 17.1) versus male SMR 6.4 (4.5, 9.0). Hence, the difference from the control pupils was greater in children rather than young people for both females and males.

- Insert Figure 1 about here -

Cause of Death

Cause of death data was available for over 95% of deaths among pupils with intellectual disabilities and over 90% deaths among controls. Table 3 shows the underlying causes of death and all-contributing causes of death by ICD 10 chapter. There were major differences between pupils with intellectual disabilities and controls with regards to the most common underlying causes. Among

For peer review only - http://bmjopen.bmj.com/site/about/guidelines.xhtml

pupils with intellectual disabilities, these were diseases of the nervous system (33%), congenital malformations, deformities and chromosomal abnormalities (22%), followed by nutritional, metabolic and endocrine diseases (8%) of which most were conditions which were the cause of the pupils' intellectual disabilities e.g. neuronal ceroid lipofuscinosis or ornithine metabolism disorders. These were followed by respiratory diseases (8%) and neoplasms (7%). The most common underlying cause of death among control pupils was deaths due to external causes (46%), which made up a higher proportion of all deaths than in the pupils with intellectual disabilities (5%). Among controls, 70% of deaths due to external causes occurred in boys, compared with 100% in the intellectual disabilities group.

There were also differences in the most common all-contributing causes of death - those with any mention on the death certificate. These chapters were not mutually exclusive, since one death could be included in several categories. Of the 106 deaths among pupils with intellectual disabilities, diseases of the nervous system contributed to 56 and diseases of the respiratory system contributed to 55. The 56 diseases of the nervous system included 34 due to cerebral palsy and 16 due to epilepsy. The 55 diseases of the respiratory system included 27 due to pneumonia, 9 due to pneumonitis associated with food and vomit, 17 due to respiratory failure, and 15 other respiratory disorders. In comparison, the control pupils had diseases of the nervous system contributing to 41 of their 461 deaths, and diseases of the respiratory system contributing to 52 of their 461 deaths which included 22 due to pneumonia. The most common all-contributing causes of death for the control pupils were external causes of morbidity and mortality in 231 of their 461 deaths (compared with 16 of 106 deaths of the pupils with intellectual disabilities), and injury, poisoning and other consequences of external causes in 219 of their 461 deaths (compared with 10 of the 106 deaths in the pupils with intellectual disabilities).

For peer review only

Table 3. The underlying causes of death and all-contributing factors in death by ICD 10 chapter for pupils with and without intellectual disabilities

	Underlying cause of death		All-contributing factors in death	
	Intellectual disabilities, n	Controls, n	Intellectual disabilities, n	Controls, n
Chapter 1: Certain Infectious and parasite diseases	<5 (5%)	12 (3%)	8	29
Chapter 2: Neoplasms	7 (7%)	92 (20%)	8	94
Chapter 3: Diseases of the blood and blood-forming organs and certain disorders involving the immune mechanism	<5 (5%)	<5 (1%)	<5	8
Chapter 4: Endocrine, nutritional and metabolic diseases	9 (8%)	15 (3%)	11	19
Chapter 5: Mental and behavioural disorders	<5 (5%)	<5 (1%)	6	17
Chapter 6: Diseases of the Nervous system	35 (33%)	20 (4%)	56	41
Chapter 8: Diseases of the Ear and mastoid process	<5 (5%)	0	<5	0
Chapter 9: Diseases of the Circulatory System	<5 (5%)	25 (5%)	15	56
Chapter 10: Diseases of the Respiratory System	8 (8%)	18 (4%)	55	52
Chapter 11: Diseases of the Digestive System	<5 (5%)	6 (1%)	13	11
Chapter 13: Diseases of the Musculoskeletal system and connective tissue	0	<5 (1%)	<5	7
Chapter 14: Diseases of the genitourinary system	<5 (5%)	<5 (1%)	<5	<5
Chapter 15: Pregnancy, childbirth and the puerperium	0	<5 (1%)	0	<5
Chapter 17: Congenital malformations, deformities and chromosomal abnormalities	23 (22%)	13 (3%)	32	18
Chapter 18: Symptoms, signs and abnormal clinical and laboratory findings, not elsewhere classified	NA	NA	28	76
Chapter 19: Injury, poisoning and certain other consequences of external causes	NA	NA	10	219
Chapter 20: External causes of morbidity and mortality	5 (5%)	210 (46%)	16	231
Unknown cause or error in underlying code	5 (5%)	39 (8%)	NA	NA
**TOTAL**	**106**	**461**	**NA**	**NA**

Note – categories under 5 were repressed due to statistical disclosure.

For peer review only - http://bmjopen.bmj.com/site/about/guidelines.xhtml

Table 4 reports these data by presenting the cause-specific crude mortality rates by ICD 10 chapter for all pupils.

For peer review only

For peer review only - http://bmjopen.bmj.com/site/about/guidelines.xhtml

Table 4. Cause-specific crude mortality rates per 100,000 person-years for pupils with intellectual disabilities and control pupils

ICD chapter[a] (Ranked by frequency in ID)	Underlying cause of death								All-contributing factors in death							
	Intellectual disabilities				Controls				Intellectual disabilities				Controls			
	(n)	CMR	95% CI		(n)	CMR	95% CI		(n)	CMR	95% CI		(n)	CMR	95% CI	
Chapter 6: Diseases of the Nervous system	(35)	51.1	36.7	71.1	(20)	0.5	0.3	0.8	(56)	81.7	62.9	106.2	(41)	1.1	0.8	1.5
Chapter 17: Congenital malformations, deformities and chromosomal abnormalities	(23)	33.6	22.3	50.5	(13)	0.4	0.2	0.6	(32)	46.7	33.0	66.0	(18)	0.5	0.3	0.8
Chapter 4: Endocrine, nutritional and metabolic diseases	(9)	13.1	6.8	25.2	(15	0.4	0.2	0.7	(11)	16.0	8.9	29.0	(19)	0.5	0.3	0.8
Chapter 10: Diseases of the Respiratory System	(8)	11.7	5.8	23.3	(18)	0.5	0.3	0.8	(55)	80.2	61.6	104.5	(52)	1.4	1.1	1.9
Chapter 20: External causes of morbidity and mortality	(5)	7.3	3.0	17.5	(210)	5.7	5.0	6.5	(16)	23.3	14.3	38.1	(231)	6.3	5.5	7.1
Chapter 9: Diseases of the Circulatory System	(<5)	Na							(15)	21.9	13.2	36.3	(56)	1.5	1.2	2.0
Chapter 11: Diseases of the Digestive System	(<5)	Na							(13)	19.0	11.0	32.7	(11)	0.3	0.2	0.5
**ICD chapter[a] *(where n<10)***																
Chapter 2: Neoplasms	(7)	10.2	4.9	21.4	(92)	2.5	2.0	3.1	(8)	11.7	5.8	23.3	(94)	2.5	2.1	3.1
Chapter 1: Certain Infectious and parasite Diseases	(<5)	Na							(8)	11.7	5.8	23.3	(29)	0.8	0.5	1.1
Chapter 5: Mental and behavioural disorders	(<5)	Na							(6)	8.8	3.9	19.5	(17)	0.5	0.3	0.7

CMR=crude mortality rate ID=intellectual disabilities

[a] ICD chapters with n<5 not reported due to statistical disclosure

For peer review only

The top 10 individual leading causes of death are shown in table 5. Among pupils with intellectual disabilities, the highest number of individual underlying cause of deaths were cerebral palsy (18%), followed by congenital brain deformities (8%), and neoplasms (7%). Where there were fewer than five individual deaths per cause, these causes were not reported due to statistical disclosure control. For the majority of deaths in pupils with intellectual disabilities this was the case; 85% of specific causes could not be disclosed. Among control pupils, the highest number of individual underlying cause of deaths were neoplasms (20%), and road traffic accidents (16%). In relation to their peers, only three of the top ten underlying causes of death among children with intellectual disabilities featured in the top ten list for the controls – neoplasms (7% vs 20% of controls), epilepsy (5% vs 2% controls), and accidents (non-road traffic related, <5% vs 9% controls).

For peer review only

For peer review only - http://bmjopen.bmj.com/site/about/guidelines.xhtml

Table 5. The top ten specific underlying causes of death, and all-contributing causes of death, for pupils with and without intellectual disabilities

Pupils with intellectual disabilities				Pupils without intellectual disabilities			
**Underlying cause of death**	**n**	**All-contributing factors**	**n**	**Underlying cause of death**	**n**	**All-contributing factors**	**n**
Cerebral palsy	19	Cerebral palsy	34	All neoplasms	92	Signs and symptoms: injury	114
Brain deformity	9	Pneumonia	27	Traffic accident	76	All neoplasms	94
All neoplasms	7	Respiratory failure	17	Self-harm	54	Traffic accidents	76
Muscular dystrophy	6	Epilepsy	16	Accidents, other	41	Self-harm	54
Epilepsy	5	Respiratory disorders	15	External, undetermined intent	25	Signs and symptoms: asphyxiation	51
Chromosomal abnormalities	5	Brain deformity	12	Asthma	14	Accident, other	43
Neuronal ceroid lipofuscinosis	<5	Chromosomal abnormalities	10	Assault	13	Signs and symptoms: poisoning	29
Pneumonia including influenza	<5	Pneumonitis due to food and vomit	9	Infections	12	All infections	29
Congenital heart disease	<5	All neoplasms	8	Epilepsy	9	External, undetermined intent	26
Accidents, other	<5	All infections	8	Cystic fibrosis	8	Pneumonia	22
Unknown causes	5	Ill-defined or ambiguous death	8	Unknown causes	39	Ill-defined or ambiguous death	58

For peer review only

For peer review only - http://bmjopen.bmj.com/site/about/guidelines.xhtml

Cause-specific SMRs, indirectly standardised using five-year age-bands, are shown in Figure 2. For underlying causes, this was only possible for the two largest categories (by ICD 10 chapters); SMR 101.4 (67.4, 152.5) for congenital malformations, deformities and chromosomal abnormalities, and SMR 89.7, (64.4, 125.0) for diseases of the nervous system. For all-contributing causes, the age-SMR for congenital malformations, deformities and chromosomal abnormalities was 97.6 (69.0, 138.0), and for diseases of the nervous system, was 71.7 (55.2, 93.2). The ratios were also high for diseases of the digestive system at 62.9 (36.5, 108.4); and for diseases of the respiratory system at 53.6 (41.2, 69.8). Despite external causes contributing to a larger proportion of deaths among the control group, the mortality rate was still higher in the intellectual disabilities group than in the controls; the crude rate was 23.3 per 100,000 person-years, compared to 6.3 per 100,000 for the controls for external cause of death (either as the underlying cause or as a contributing factor). This produced an SMR of 3.5 (2.2, 5.8) demonstrating there is considerable over-representation in the intellectual disabilities group versus the controls.

- Insert Figure 2 about here -

Avoidable mortality

According to the UK ONS definition of avoidable mortality, (deaths which are amenable, preventable, or both), 19% of deaths in the intellectual disabilities cohort were classed as avoidable; 15% of deaths were amenable to treatment, and 6% were preventable. The majority of avoidable deaths (80%) were considered amenable to treatment, for their age group, including epilepsy, pneumonia and neoplasms. Among the control pupils, 63% of deaths were classed as avoidable, 16% were amenable to treatment, and 48% were preventable. As recommended by the ONS[22], avoidable mortality rates based on low numbers should be labelled as unreliable and marked “U” or ^{U}. The crude avoidable mortality rate for pupils with intellectual disabilities was higher, at 29.2^{U} (18.9, 45.2) per 100,000 in pupils with intellectual disabilities, compared to 7.9 (7.0, 8.8) per 100,000 in the control pupils. The SMR was 3.5^{U} (2.3, 5.4). Further breakdown of avoidable rates was possible for deaths that were amenable to healthcare; in the intellectual disabilities group, the amenable mortality rate was 23.3^{U} (14.3, 38.1) per 100,000 versus 2.0^{U} (1.6, 2.6) per 100,000 in controls; and the SMR was found to be 10.9^{U}, (6.7, 17.8).

Among pupils with intellectual disabilities, there were additional causes of death that the authors of this paper consider would have been amenable to health care: aspiration pneumonia; otitis media; megacolon; gastrointestinal haemorrhage; gastroenteritis; and contributing causes of death including gastro-oesophageal reflux, and urinary tract infections. These are not currently included within the ONS list of underlying causes.

For peer review only - http://bmjopen.bmj.com/site/about/guidelines.xhtml

Discussion

Principle findings and interpretation

Our study is one of very few that has reported mortality rates among children and young people with intellectual disabilities, and is highly novel in reporting underlying, all-contributing, and the most common individual causes of death at this age, including cause-specific SMRs. We have demonstrated that children and young people in Scotland with intellectual disabilities have a 12-fold risk of death compared to their peers, rising to 21-fold on excluding external causes. Pupils with intellectual disabilities were also over-represented in deaths that were amenable to healthcare, and were approximately 3.5 times more likely to experience an avoidable death (albeit calculated using unreliably low rates). Children aged 5 -14 years with intellectual disabilities had a higher risk relative to peers (SMR 22.5), than the young people aged ≥15 years with intellectual disabilities (SMR 8.1). This difference reflects that, in the general population, there were considerably more deaths in young people than in children, especially for males, as opposed to more deaths of children than young people with intellectual disabilities. The SMR was higher for female pupils in both age groups, reflecting the higher death rate of males in the controls. Nervous system and respiratory causes of death were the most common among children and young people with intellectual disabilities, including deaths that would have been amenable to quality health care, such as epilepsy, pneumonia, and pneumonitis due to food and vomit. It is highly important to identify amenable deaths so that actions can be devised and taken. Causes of death among children and young people with intellectual disabilities were higher across several disease categories than for other children and young people, including diseases of the nervous system, digestive system, respiratory system, endocrine, nutrition and metabolic diseases, diseases of the circulatory system, and external causes.

Previous studies have demonstrated that there is an increased risk of sudden unexpected death in epilepsy among people with intellectual disabilities, however, in our study, the majority of deaths which listed epilepsy as a contributing factor, also listed pneumonia, so this does not appear to account for our findings.

Whilst external causes of deaths accounted for the greatest proportion of deaths among control children and young people (46%), especially in males, we found that external causes of death were still over-represented among children and young people with intellectual disabilities compared with their controls (partly due to inhalation of gastric contents, and inhalation of objects obstructing breathing). Trollor et al. (2017)[19] hypothesised that higher SMRs in adult women than men with intellectual disabilities may be driven by the larger proportion of male deaths in the general population due to external causes; and the lack of equivalent deaths in males with intellectual disabilities.

For peer review only - http://bmjopen.bmj.com/site/about/guidelines.xhtml

However, in our population of children and young people, when we re-calculated SMRs to exclude external causes, the observed increase in risk for females remained. Hence at this age range, this is only a partial explanation for the sex differences in SMRs, and there are further risk factors and vulnerabilities which require further exploration. It should be noted however, that in children and young people with intellectual disabilities, not all studies report a higher SMR in females compared with males[10, 11].

Comparison with previous studies

Two previous studies[9, 16] have reported a higher SMR for children than for young people. Glover et al. 2017[9] reported results separately for children aged 0-9 years, (SMR 30.4) and young people aged 10-17 years with intellectual disabilities (SMR 17.3). The Australian study by Bourke et al. 2017[16] reported a higher adjusted Hazard Ratio (aHR) for children aged 6-10 years (aHR 12.6) than young people aged 11-25 years (aHR 4.9). The SMRs we report are lower than those reported by Glover et al. (2017)[9], but the extent of difference between the children and young people is similar, albeit for differently defined age groups. The confidence intervals reported in our study are narrower due to the larger sample size. The SMRs we report are higher than those previously reported from small scale studies in Finland and USA[8, 10, 14], and a larger one in the Republic of Ireland[15], yet lower than a study reported from England[9], and a small study in Canada[11]. These differences may be due to actual international differences, or due to methodological differences between studies including: the method and source of identification of the population with intellectual disabilities; age ranges included; and study size with several of the previous studies having produced results with wide confidence intervals. All of these studies report a higher SMR in females than in males, except the study conducted in Canada and one in Finland.

The only previous study that has reported cause of death for children and young people did so for the age group 1-25 years, so includes some deaths of adults rather than just children and young people[16]. It reported the most common causes of death to be infections in 50% (particularly respiratory infections in 34%), birth defects in 19% (particularly cardiac defect in 15%), and accidents in 11%, although by ICD 10 chapter deaths due to diseases of the respiratory tract were reported for 4.6%, infections and parasitic diseases were 3.1%, and external causes were 7.7%; and the most common were congenital malformations, deformities and chromosomal abnormalities in 29.1%, and diseases of the nervous system in 27.6%. They did not report cause-specific SMRs by ICD 10 chapters, but crude numbers were proportionally higher for the children with intellectual disabilities for diseases of the nervous system, endocrine, nutritional and metabolic diseases, and congenital malformations, and lower for conditions originating in the perinatal period, external causes, or injury

For peer review only - http://bmjopen.bmj.com/site/about/guidelines.xhtml

or poisoning[16]. We demonstrated diseases of the nervous system and respiratory system to be the most common causes of death, and that cause-specific SMRs were raised across all of congenital malformations, deformities and chromosomal abnormalities, diseases of the nervous system, digestive system, respiratory system, endocrine, nutrition and metabolic diseases, circulatory system, and external causes.

Glover et al (2017) graphed avoidable deaths in his study of children and adults[9]. We are unaware of any previous studies numerically quantifying amenable deaths among children and young people with intellectual disabilities.

Strengths and limitations

Our study drew upon data from an entire country, collected annually, and linked to national death records. It was large in scale, including over 18,000 children and young people with intellectual disabilities, and a large control population. A record of intellectual disabilities at school brings an entitlement to additional support so is likely to drive good recording in high income countries like Scotland. However, it only uses a binary definition for intellectual disabilities, therefore the study could not investigate mortality among people with different causes and severities of intellectual disabilities. Our study was not large enough to delineate cause-specific mortality ratios by sex, nor to study whether there are any ethnic variations. Use of death certificate data is known to have limitations[22], including inconsistent reporting and no reporting of severity of conditions. There may be some diagnostic overshadowing in death certificate data for people with intellectual disabilities, obscuring the events leading to death[23-25]. The ONS list of avoidable deaths does not include some that appear important among children and young people with intellectual disabilities, such as aspiration pneumonia, otitis media, megacolon, gastrointestinal haemorrhage, gastroenteritis, which featured as an underlying cause of death in our data. Additionally, death certificate data does not include wider determinants of health and death that may be implicated, such as being the target of discrimination or neglect.

Conclusions and future directions

It is extremely important to study deaths among children and young people with intellectual disabilities, especially as so few studies have previously done so. Amongst the studies that have, there exists wide variation in the extent of reported inequality compared to other children and young people, and wide confidence intervals, but all show a higher SMR. Our large study provides robust data that quantifies the extent of the difference; children and young people have a 12 times higher risk of death. A larger body of research exists for adults (rather than children and young people) with

For peer review only - http://bmjopen.bmj.com/site/about/guidelines.xhtml

intellectual disabilities, and demonstrates substantial inequalities, and a high proportion of amenable deaths that could be addressed via reasonable adjustments in care provision. In our study, we have now reported that children and young people with intellectual disabilities also experience inequalities and experience amenable deaths. This is important, and we need a better understanding of it so that targeted improvements in care can start to be developed and delivered to reduce this inequality. Heslop et al (2014)[26] conducted a confidential inquiry into deaths of people with intellectual disabilities and made recommendations for improvements to practice regarding respiratory deaths, including aggressive monitoring and treatment of gastro-oesophageal reflux as well as postural and physical therapies. We have found that this is also important for children and young people with intellectual disabilities, if we are serious about improving life expectancy. Additionally, Scotland now offers influenza vaccines to all primary school aged children to reduce pneumonia; we therefore need to understand uptake by children with intellectual disabilities, and its determinants, to gauge whether this will change mortality findings.

The results of this study should be used to inform and direct multidisciplinary healthcare teams, as well as educators and carers to the associated risks of mortality in childhood and generate greater awareness around potential areas of improvement. Our countrywide study had a mean follow-up of around 5 years, and given that the pupil census is recorded annually, it presents the framework for further work to investigate both mortality trends in children and young people with intellectual disabilities, and a more detailed understanding of these.

Acknowledgements: We thank the ScotXed Pupil Census, the National Records of Scotland, and Information Services Division (ISD) of NHS Scotland for providing data and the eData Research and Innovation Service team at ISD Scotland for assisting with the data linkage and analysis stage of this project.

Contributors: GS analysed the data, interpreted findings and wrote the first draft of the manuscript. MF developed record linkage, analysed the data, interpreted findings and contributed to the manuscript. JP developed record linkage, interpreted findings and contributed to the manuscript. DK, AH & CM interpreted data and contributed to the manuscript, S-AC conceived the study, analysed and interpreted the data, and contributed to the manuscript. All authors approved the final version of the manuscript.

Funding: This study was funded by an MRC Mental Health Data Pathfinder Award (MC_PC_17217), and the Scottish Government via the Scottish Learning Disabilities Observatory.

Disclaimer: The funders had no role in the study design, collection, analyses or interpretation of data, writing the report nor the decision to submit the article for publication.

For peer review only - http://bmjopen.bmj.com/site/about/guidelines.xhtml

Competing Interests: None declared.

Patient Consent: Not required.

Ethics approval: This study received approval from the NHS National Services Scotland Privacy Advisory Committee.

Data sharing statement: This study linked patient information held across several administrative health datasets within ISD Scotland, with externally held data held by the Scottish Government (ScotXed education), and National Records of Scotland. Linkage and de-identification of data was performed by the Information Services Division (ISD) of NHS National Services Scotland (NSS). A data processing agreement between NHS NSS and University of Glasgow and a data sharing agreement between ScotXed and University of Glasgow was drafted. The University of Glasgow were authorised to receive record linked data controlled and held by ISD within NSS, via access through the national safe haven. The ISD Statistical Disclosure Control Protocol was followed. It is therefore not possible to share data with other parties.

For peer review only

For peer review only - http://bmjopen.bmj.com/site/about/guidelines.xhtml

References

1. Emerson E, Hatton C. Mental health of children and adolescents with intellectual disabilities in Britain. *Br J Psychiatry* 2007;191:493-499.

2. Hughes-McCormack LA, Rydzewska E, Henderson A, et al. Prevalence and general health status of people with intellectual disabilities in Scotland -a total population study. *J Epidemiol Community Health* 2017a;72:78–85.

3. Hughes-McCormack LA, Rydzewska E, Henderson A, et al. Prevalence of mental health conditions and relationship with general health in a whole country population of people with intellectual disabilities compared with the general population. *BJPsych Open* 2017b;3:243-248.

4. Heslop P, Lauer E, Hoghton M. Mortality in people with intellectual disabilities. *J Appl Res Intellect Disabil* 2015;28:367–372.

5. Hosking FJ, Carey IM, Shah SM, et al. Mortality among adults with intellectual disability in England: Comparisons with the general population. *Am J Public Health* 2016;106(8):1483–1490

6. O'Leary L, Cooper SA, Hughes-McCormack LA. Early death and causes of death of people with intellectual disabilities: A systematic review. *J Appl Res Intellect Disabil* 2018a;31:325–342.

7. O'Leary L, Hughes-McCormack LA, Dunn K, et al. Early death and causes of death of people with Down syndrome a systematic review. *J Appl Res Intellect Disabil* 2018b;31(5):687-708

8. Decouflé P, Autry A. Increased mortality in children and adolescents with developmental disabilities. *Paediatr Perinat Epidemiol* 2002;16(4):375–382

9. Glover G, Williams R, Heslop P, et al. Mortality in people with intellectual disabilities in England. *J Intellect Disabil Res* 2017;61(1):62–74

10. Patja K, Mölsä P, Iivanainen M. Cause-specific mortality of people with intellectual disability in a population-based, 35-year follow-up study. *J Intellect Disabil Res* 2001;45:30–40

11. Ouellette-Kuntz H, Shooshtari S, Balogh R, et al. Understanding information about mortality among people with intellectual and developmental disabilities in Canada. *J Appl Res Intellect Disabil* 2015;28(5):423–435

12. McGuigan SM, Hollins S, Attard M. Age-specific standardized mortality rates in people with learning disability. *J Intellect Disabil Res* 1995;39(6):527-531

13. Forsgren L, Edvinsson SO, Nystrom L, et al. Influence of epilepsy on mortality in mental retardation: An epidemiologic study. *Epilepsia* 1996;37(10):956–963.

14. Arvio M, Salokivi T, Tiitinen A, et al. Mortality in individuals with intellectual disabilities in Finland. *Brain Behav* 2016;6(2):1–4.

15. McCarron M, Carroll R, Kelly C, et al. Mortality rates in the general Irish population compared to those with an intellectual disability from 2003 to 2012. *J Appl Res Intellect Disabil* 2015;28(5):406–413

For peer review only - http://bmjopen.bmj.com/site/about/guidelines.xhtml

16. Bourke J, Nembhard WN, Wong K, et al. Twenty-Five Year Survival of Children with Intellectual Disability in Western Australia. *J Pediatr* 2017;188:232-239.e2.

17. Durvasula S, Beange H, Baker W. Mortality of people with intellectual disability in northern Sydney. *J Intellect Dev Disabil* 2002;27(4):255–264.

18. Scottish Index of Multiple Deprivation Background and Methodology, Scottish Government Website. https://www2.gov.scot/Topics/Statistics/SIMD/BackgroundMethodology (accessed July 2019)

19. Trollor J, Srasuebkul P, Xu H, et al. Cause of death and potentially avoidable deaths in Australian adults with intellectual disability using retrospective linked data. *BMJ Open* 2017;7:e013489.

20. International Statistical Classification of Diseases and Related Health Problems 10th Revision, Volume 2 Instruction manual, 2010 edition. WHO. https://www.who.int/classifications/icd/ICD10Volume2_en_2010.pdf (accessed July 2019).

21. Olatunde O, Windsor-Shellard B, Campbell A. Revised definition of avoidable mortality and new definition for children and young people. Office for National Statistics 2016. https://www.ons.gov.uk/file?uri=/aboutus/whatwedo/statistics/consultationsandsurveys/allconsultationsandsurveys/reviewofavoidablemortalitydefinition/reviseddefinitionofavoidablemortalityandnewdefinitionforchildrenandyoungpeople.doc (accessed July 2019)

22. Office of National Statistics: Avoidable mortality in the UK Quality and Methodology Information. Office of National Statistics 2019. https://www.ons.gov.uk/peoplepopulationandcommunity/healthandsocialcare/causesofdeath/methodologies/avoidablemortalityinenglandandwalesqmi (accessed July 2019)

23. Landes SD, Peek CW. Death by mental retardation? The influence of ambiguity on death certificate coding error for adults with intellectual disability. *J Intellect Disabil Res* 2013;57(12):1183-1190

24. Glover, G, Ayub M. How people with learning disabilities die. Improving Health and Lives Learning Disability Observatory, Durham 2010. https://www.gov.uk/government/publications/people-with-learning-disabilities-in-england-2015 (accessed July 2019)

25. Landes SD, Stevens JD, Turk MA. Obscuring effect of coding developmental disability as the underlying cause of death on mortality trends for adults with developmental disability: a cross-sectional study using US Mortality Data from 2012 to 2016. *BMJ Open* 2019;9:e026614.

26. Heslop P, Blair PS, Fleming P, et al. The Confidential Inquiry into premature deaths of people with intellectual disabilities in the UK: a population-based study. *Lancet* 2014;383(9920):889-895

For peer review only

For peer review only - http://bmjopen.bmj.com/site/about/guidelines.xhtml

Figure 1. Forest Plot of age-standardised and gender-specific mortality ratios for pupils with intellectual disabilities

Figure 2. Forest plot of cause-specific age-Standardised Mortality Ratios for pupils with intellectual disabilities by ICD 10 chapter for underlying cause of death and for all-contributing factors of death

Footnote: Age-standardised mortality ratios (SMRs) & 95% confidence intervals were calculated using 5-year age bands for all ICD 10 chapters with ≥10 deaths

For peer review only

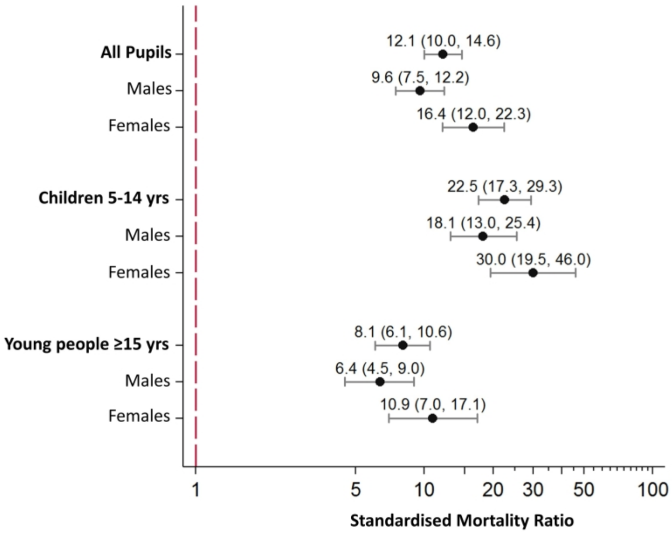

Figure 1. Forest Plot of age-standardised and gender-specific mortality ratios for pupils with intellectual disabilities

338x190mm (300 x 300 DPI)

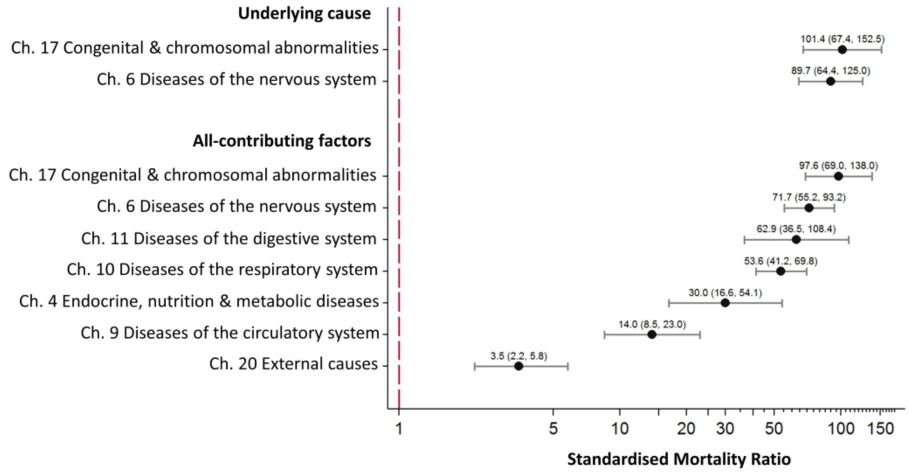

Figure 2. Forest plot of cause-specific age-Standardised Mortality Ratios for pupils with intellectual disabilities by ICD 10 chapter for underlying cause of death and for all-contributing factors of death
Footnote: Age-standardised mortality ratios (SMRs) & 95% confidence intervals were calculated using 5-year age bands for all ICD 10 chapters with ≥10 deaths

338x190mm (300 x 300 DPI)

STROBE Statement—checklist of items that should be included in reports of observational studies

	Item No	Recommendation	Page No
**Title and abstract**	1	(*a*) Indicate the study's design with a commonly used term in the title or the abstract	1
		(*b*) Provide in the abstract an informative and balanced summary of what was done and what was found	1
**Introduction**			
Background/rationale	2	Explain the scientific background and rationale for the investigation being reported	3
Objectives	3	State specific objectives, including any prespecified hypotheses	5
**Methods**			
Study design	4	Present key elements of study design early in the paper	6
Setting	5	Describe the setting, locations, and relevant dates, including periods of recruitment, exposure, follow-up, and data collection	6
Participants	6	(*a*) *Cohort study*—Give the eligibility criteria, and the sources and methods of selection of participants. Describe methods of follow-up<br>*Case-control study*—Give the eligibility criteria, and the sources and methods of case ascertainment and control selection. Give the rationale for the choice of cases and controls<br>*Cross-sectional study*—Give the eligibility criteria, and the sources and methods of selection of participants	6
		(*b*) *Cohort study*—For matched studies, give matching criteria and number of exposed and unexposed<br>*Case-control study*—For matched studies, give matching criteria and the number of controls per case	Na
Variables	7	Clearly define all outcomes, exposures, predictors, potential confounders, and effect modifiers. Give diagnostic criteria, if applicable	6
Data sources/ measurement	8*	For each variable of interest, give sources of data and details of methods of assessment (measurement). Describe comparability of assessment methods if there is more than one group	*7*
Bias	9	Describe any efforts to address potential sources of bias	7
Study size	10	Explain how the study size was arrived at	5
Quantitative variables	11	Explain how quantitative variables were handled in the analyses. If applicable, describe which groupings were chosen and why	6
Statistical methods	12	(*a*) Describe all statistical methods, including those used to control for confounding	6
		(*b*) Describe any methods used to examine subgroups and interactions	7
		(*c*) Explain how missing data were addressed	Na
		(*d*) *Cohort study*—If applicable, explain how loss to follow-up was addressed<br>*Case-control study*—If applicable, explain how matching of cases and controls was addressed<br>*Cross-sectional study*—If applicable, describe analytical methods taking account of sampling strategy	Na
		(*e*) Describe any sensitivity analyses	Na

Continued on next page

For peer review only - http://bmjopen.bmj.com/site/about/guidelines.xhtml

**Results**			
Participants	13*	(a) Report numbers of individuals at each stage of study—eg numbers potentially eligible, examined for eligibility, confirmed eligible, included in the study, completing follow-up, and analysed	7
		(b) Give reasons for non-participation at each stage	8
		(c) Consider use of a flow diagram	Na
Descriptive data	14*	(a) Give characteristics of study participants (eg demographic, clinical, social) and information on exposures and potential confounders	8
		(b) Indicate number of participants with missing data for each variable of interest	Na
		(c) *Cohort study*—Summarise follow-up time (eg, average and total amount)	10
Outcome data	15*	*Cohort study*—Report numbers of outcome events or summary measures over time	10
		Case-control study—Report numbers in each exposure category, or summary measures of exposure	
		Cross-sectional study—Report numbers of outcome events or summary measures	
Main results	16	(*a*) Give unadjusted estimates and, if applicable, confounder-adjusted estimates and their precision (eg, 95% confidence interval). Make clear which confounders were adjusted for and why they were included	10
		(*b*) Report category boundaries when continuous variables were categorized	10
		(*c*) If relevant, consider translating estimates of relative risk into absolute risk for a meaningful time period	
Other analyses	17	Report other analyses done—eg analyses of subgroups and interactions, and sensitivity analyses	17
**Discussion**			
Key results	18	Summarise key results with reference to study objectives	18
Limitations	19	Discuss limitations of the study, taking into account sources of potential bias or imprecision. Discuss both direction and magnitude of any potential bias	20
Interpretation	20	Give a cautious overall interpretation of results considering objectives, limitations, multiplicity of analyses, results from similar studies, and other relevant evidence	20
Generalisability	21	Discuss the generalisability (external validity) of the study results	20
**Other information**			
Funding	22	Give the source of funding and the role of the funders for the present study and, if applicable, for the original study on which the present article is based	21

*Give information separately for cases and controls in case-control studies and, if applicable, for exposed and unexposed groups in cohort and cross-sectional studies.

Note: An Explanation and Elaboration article discusses each checklist item and gives methodological background and published examples of transparent reporting. The STROBE checklist is best used in conjunction with this article (freely available on the Web sites of PLoS Medicine at http://www.plosmedicine.org/, Annals of Internal Medicine at http://www.annals.org/, and Epidemiology at http://www.epidem.com/). Information on the STROBE Initiative is available at www.strobe-statement.org.

For peer review only

BMJ Open

Rates and causes of mortality among children and young people with and without intellectual disabilities in Scotland: a record linkage cohort study of 796,190 schoolchildren

Journal:	*BMJ Open*
Manuscript ID	bmjopen-2019-034077.R1
Article Type:	Original research
Date Submitted by the Author:	08-Apr-2020
Complete List of Authors:	Smith, Gillian; University of Glasgow Institute of Health and Wellbeing<br>Fleming, Michael ; University of Glasgow, Institute of Health and Wellbeing<br>Kinnear, Deborah; University of Glasgow, Institute of Health and Wellbeing<br>Henderson, Angela; University of Glasgow, Institute of Health and Wellbeing<br>Pell, J. P.; University of Glasgow, Institute of Health and Wellbeing<br>Melville, Craig; University of Glasgow, Institute of Health and Wellbeing<br>Cooper, Sally-Ann; University of Glasgow, Institute of Health and Wellbeing
**Primary Subject Heading**:	Epidemiology
Secondary Subject Heading:	Public health, Paediatrics, Mental health
Keywords:	intellectual disabilities, mortality, death, children, young people

SCHOLARONE™ Manuscripts

I, the Submitting Author has the right to grant and does grant on behalf of all authors of the Work (as defined in the below author licence), an exclusive licence and/or a non-exclusive licence for contributions from authors who are: i) UK Crown employees; ii) where BMJ has agreed a CC-BY licence shall apply, and/or iii) in accordance with the terms applicable for US Federal Government officers or employees acting as part of their official duties; on a worldwide, perpetual, irrevocable, royalty-free basis to BMJ Publishing Group Ltd ("BMJ") its licensees and where the relevant Journal is co-owned by BMJ to the co-owners of the Journal, to publish the Work in this journal and any other BMJ products and to exploit all rights, as set out in our licence.

The Submitting Author accepts and understands that any supply made under these terms is made by BMJ to the Submitting Author unless you are acting as an employee on behalf of your employer or a postgraduate student of an affiliated institution which is paying any applicable article publishing charge ("APC") for Open Access articles. Where the Submitting Author wishes to make the Work available on an Open Access basis (and intends to pay the relevant APC), the terms of reuse of such Open Access shall be governed by a Creative Commons licence – details of these licences and which Creative Commons licence will apply to this Work are set out in our licence referred to above.

Other than as permitted in any relevant BMJ Author's Self Archiving Policies, I confirm this Work has not been accepted for publication elsewhere, is not being considered for publication elsewhere and does not duplicate material already published. I confirm all authors consent to publication of this Work and authorise the granting of this licence.

For peer review only

Rates and causes of mortality among children and young people with and without intellectual disabilities in Scotland: a record linkage cohort study of 796,190 schoolchildren

Smith, G.S., Fleming, M., Kinnear, D., Henderson, A., Pell, J.P., Melville, C., Cooper, S-A.

Corresponding author: Professor Sally-Ann Cooper; Sally-Ann.Cooper@glasgow.ac.uk

For peer review only

Abstract

Objectives: To investigate mortality rates and causes in children and young people with intellectual disabilities.

Design: Retrospective cohort; individual record-linkage between Scotland's annual pupil census and National Records of Scotland death register.

Setting: General community.

Participants: Pupils receiving Local Authority-funded schooling in Scotland, 2008-2013, with an Additional Support Need due to intellectual disabilities, compared with other pupils.

Main outcome measures: Deaths up to 2015: age of death, sex- and age-standardised mortality ratios (SMRs); causes of death including cause-specific age-SMRs; avoidable deaths as defined by UK Office of National Statistics.

Results: 18,278/947,922 (1.9%) pupils had intellectual disabilities. 106 died over 67,342 person-years (crude mortality rate=157/100,000 person-years), compared with 458 controls over 3,672,224 person-years (crude mortality rate=12/100,000 person-years). Age-, sex-SMR was 11.6 (95% CI 9.6, 14.0); 16.6 (12.8, 22.6) for female pupils, 9.8 (7.7, 12.5) for male pupils. Most common main underlying causes were diseases of the nervous system, then congenital anomalies; most common all-contributing causes were diseases of the nervous system, then respiratory system; most common specific contributing causes were cerebral palsy, pneumonia, respiratory failure, and epilepsy. For all contributing causes, SMR was 98.8 (69.9, 139.7) for congenital anomalies, 76.5 (58.9, 99.4) for nervous system, 63.7 (37.0, 109.7) for digestive system, 55.3 (42.5, 72.1) for respiratory system, 32.1 (17.8, 57.9) for endocrine, 14.8 (8.9, 24.5) for circulatory system. External causes accounted for 46% of

For peer review only - http://bmjopen.bmj.com/site/about/guidelines.xhtml

control deaths, but the SMR for external-related deaths was still higher (3.6 {2.2, 5.8}) for pupils with intellectual disabilities. Deaths amenable to good care were common.

Conclusions: Pupils with intellectual disabilities were much more likely to die than their peers, and had a different pattern of causes, including amenable deaths across a wide range of disease categories. Improvements are needed to reduce amenable deaths e.g. epilepsy-related and dysphagia, and to support families of children with life-limiting conditions.

Strengths and limitations of this study

- Novel use of education records and record linkage to death records to study mortality in an unselected cohort of children and young people with intellectual disabilities
- Due to the use of a whole country population these results are well-powered and generalisable
- Despite comprising a whole country population, our study was not large enough to delineate cause-specific mortality ratios by sex
- This study was limited by lack of demographic and clinical diagnostic information including the severity or cause of intellectual disabilities
- Reliance on death certificate data is limited by inconsistencies in reporting of cause of death

For peer review only

Key words

Intellectual disabilities, mortality, death, children, young people

For peer review only - http://bmjopen.bmj.com/site/about/guidelines.xhtml

Introduction

Children and young people with intellectual disabilities have a much higher prevalence of physical and mental ill-health compared to the general population[1-3]. The life expectancy of people with intellectual disabilities has been reported to be about 20 years shorter than in the general population, or 28 years shorter specifically for people with Down syndrome[4-7]. While the actual number of deaths in childhood is smaller than in adults, mortality studies comparing people with intellectual disabilities with the general population have tended to show increased risk ratios in younger age groups compared to adults. However, the excess reported risk varies considerably between studies, and not all studies are comparable due to e.g. reporting deaths within different age ranges, and additionally some have small sample sizes and wide confidence intervals. Reported standardised mortality ratios (SMR) comparing people with and without intellectual disabilities, have ranged from 3.3 (95% confidence interval [CI] 2.1, 5.0) in young people aged 10-19 years[8], to 17.3 (95% CI 9.4, 29.0) in young people aged 10-17 years[9]; from 2.6 in males aged 2-19 years and 1.7 in females aged 2-19 years[10], to 21.6 (95% CI 10.8, 38.7) in males aged 0-19 years and 18.1 (95% CI 3.7, 53.0) in females aged 0-19 years[11], and have been reported to be 30.4 (95% CI 18.4, 47.5) in children aged 0-9 years[9]. We have summarised all previous studies to our knowledge which report mortality ratios for children and young people under aged 25, with and without intellectual disabilities, where they are reported separate from older age groups (online supplementary Appendix 1).

Most of these studies do not report causes of death among children and young people with intellectual disabilities. Bourke et al. (2017)[12] reported the most common causes of death in children, young people, and adults with intellectual disabilities aged 1-25 years to be respiratory infection (34%), with an additional 10% having an aspiration related cause, congenital heart defects (15%), and accidents (11%). Compared to children and young people who did not have intellectual disabilities, their causes of death by ICD 10 chapter were more likely to be attributed to the nervous system, endocrine, nutritional and metabolic diseases, or congenital malformations, and less likely to be attributed to conditions originating in the perinatal period, external causes, or injury or poisoning[12]. Patja et al. (2001)[10] reported respiratory diseases to be the most common underlying/immediate cause of death in children and young people with intellectual disabilities aged 2-19 years, with a relative risk of 5.8 (95% CI 4.4, 15.6) in males and 4.3 (0.3, 4.7) in females, and did not find any other causes (infectious diseases, tumours, vascular diseases, diseases of digestive system, accidents and poisonings, or other causes) to differ from those expected in the general population. However, the study was limited by small sample size. Durvasula et al. (2009)[13] reported 7 of 14 deaths among

For peer review only - http://bmjopen.bmj.com/site/about/guidelines.xhtml

young people with intellectual disabilities aged 10-24 years were attributed to the respiratory system (pneumonia, aspiration).

Adults with intellectual disabilities are over-represented in deaths which would have been amenable to treatment by timely and effective health care[4-5, 9]. However, there is limited evidence on whether children and young people with intellectual disabilities also experience such amenable deaths more commonly than other children and young people, as most authors who have reported cause-specific mortality do so grouped across all ages, due to sample sizes.

Overall, as shown in Appendix 1 (online supplementary), studies on mortality in children with intellectual disabilities are mostly small in size, and results are variable. Studies of causes of death exclusively in children and young people with intellectual disabilities are also limited. Hence, the aim of this cohort study is to compare all-cause and cause-specific mortality in Scotland's school attending population with and without intellectual disabilities.

For peer review only

For peer review only - http://bmjopen.bmj.com/site/about/guidelines.xhtml

Methods

We used education data from Scotland's annual pupil census between 2008 and 2013, to establish a cohort of children and young people with and without intellectual disabilities. We used individual record linkage based on probabilistic record matching (on date of birth, sex, and postcode,) to the Community Health Index, Scotland's list of all unique patient identifiers, including the National Records for Scotland (NRS) deaths registry, to ascertain all deaths up to February 2015 in Scotland.

The Scottish annual pupil census is completed in September each year and provides information on all children attending Local Authority funded primary, secondary, and special schools in Scotland, or funded placements in alternative schools, which includes 95% of the entire population of children and young people in Scotland. This information includes whether the child has a record of Additional Support Needs, and the type of Additional Support Need. It is held by the Scottish exchange of education data (ScotXed).

The record linkage methodology required date of birth, sex and postcode, however since names were not used to link pupil records to the health data, we excluded non-singleton births (available for Scottish-born pupils only, identified from linkage to maternity records). Unlikely matches were excluded and the most likely match was selected as the correctly linked pupil record. We also excluded any records with duplicate pupil records or where the linkage was tied with another patient. We included in the study all pupils with records of Additional Support Need due to intellectual disabilities between 2008 and 2013, between the ages 4 and 19 years old, upon entry. Pupils were also censored upon reaching aged 25 if they reached this age during the observation period, so that the maximum follow-up age was 24 years old. Only pupils with intellectual disabilities recorded in at least two different school years were included in the intellectual disabilities group, to ascertain that they were correctly identified. Pupils who were included in at least two pupil censuses over the study period and had no record of intellectual disabilities or autism were used as the comparison group. Pupils with solely autism were also excluded from controls, to eliminate potential mislabelling of support need for either autism or learning disability in the absence of clinical diagnoses.

The pupil census also includes data on age, sex, ethnicity and Scottish Index of Multiple Deprivation 2012 (SIMD)[14]. Derived from individual pupil postcode of residence, SIMD is a composite of seven indices to indicate the extent of neighbourhood deprivation. SIMD was divided into quintiles according to the general population. Data on disability requirements including physical (e.g. visual, hearing, or physical impairments), communication, or curriculum needs are also listed.

For peer review only - http://bmjopen.bmj.com/site/about/guidelines.xhtml

Non-modifiable descriptive data on sex, ethnicity and SIMD, were taken from each pupils' first year in the census. For disability requirements, all records across multiple pupil census years were used to define whether having ever received adaptation requirements. Explorative statistical analysis using t-tests and χ^2-tests were employed to investigate characteristics of pupils with intellectual disabilities compared to their peers in the comparison group. Differences in age of death were explored using t-tests. Crude mortality rates were calculated using the censor date, 13 February 2015 or date of death. Since only those pupils who attended school in at least two years over our observed study period were eligible, the period between the first and second record introduced an immortal time bias, where no deaths could have occurred, and therefore the entry to the study was defined as the date of their second pupil census record. For indirect standardisation, observed deaths were assumed to be independent and vary with the Poisson distribution. The mortality rates were indirectly standardised for both males and females using the expected age-specific mortality rates per one-year age-group, using STATA's "strate" command, to calculate age- and sex-standardised mortality ratios (SMRs) for pupils with versus without intellectual disabilities. 95% confidence intervals were calculated based on the quadratic approximation of the log likelihood. Expected rates were calculated using fixed age and sex-specific rates, from the large control population. The SMRs were subsequently calculated stratified by age, into childhood (aged 5-14 years) and young people (aged ≥15 years), and by sex. The SMRs were also calculated for all deaths excluding from external causes. This was to investigate whether the over-representation of female deaths in people with intellectual disabilities compared to the general population[12, 15-16] is related to the large proportion of male deaths from external causes in the general population[17].

For all-cause mortality, Kaplan-Meier survival curves were plotted for the overall time period for both groups. Cox-proportional hazards models are also presented, adjusted for age and sex.

For cause of death analyses, the underlying cause of death is defined internationally[18] as the disease or injury which initiated the chain of morbid events leading directly to death, or the accident/act which produced the fatal injury. We also used a broader definition to analyse all-contributing causes, that included all deaths, with any mention on the death certificate related to the cause; combining both the underlying cause, with secondary, or contributing factors. While the same ICD 10 codes are used, it is important to note that one death may have several other, additional causes as contributing factors, all of which are counted in figures reporting "all-contributing causes".

For the underlying causes of death, the total number of deaths in each ICD 10 chapter were collated, and this was then repeated for specific causes listed within chapters. Any errors or ambiguous deaths were listed as an unknown cause. All deaths where the underlying cause was ill-defined; defined by

For peer review only - http://bmjopen.bmj.com/site/about/guidelines.xhtml

ICD 10 WHO guidelines[18] as codes in Chapter 18 excluding R95, were also re-classified as "unknown". Next, the breakdown of all-contributing causes were analysed, by collating number of deaths in each ICD 10 chapter. For cause-specific SMRs, indirect age-standardisation was also performed, but using expected rates per 5-year age-bands to age-standardise rates and robust standard errors were used. For categories which had fewer than ten deaths, no calculation was attempted due to lack of reliability in the small number of deaths. Furthermore, in keeping with the ONS mortality methodology[19], all mortality rates based on between ten and twenty deaths were labelled as unreliable. The Office of National Statistics (ONS) revised definition of avoidable mortality for children and young people[20] defined avoidable mortality as either amenable mortality (avoidable through good quality healthcare even after a condition has developed) or preventable mortality (avoidable through incidence reduction via public health interventions) or both. This list of ICD 10 causes was used to determine the occurrence of avoidable deaths. The rates and age-standardised mortality ratios (age-standardised using 5-year age-bands) for avoidable, amenable, and preventable mortality were calculated using robust errors, except where there were fewer than ten deaths per chapter. In keeping with the ONS avoidable mortality methodology[19], all mortality rates based on fewer than twenty deaths were labelled as unreliable.

Sensitivity analysis

A sensitivity analysis was carried out using wider inclusion criteria from the education data for both groups; the intellectual disabilities group included all pupils with at least one record of support at school due to intellectual disabilities. The control group included all pupils with at least one census record, and without support records for intellectual disabilities or autism. There were no other methodological changes made to age standardising process or censor dates, but entry date was changed to the date of the first record of support need for pupils with intellectual disabilities or the first census date for pupils without intellectual disabilities.

All statistical analyses were undertaken using Stata, version 15.0 (StataCorp).

Personal and Patient involvement

This study was undertaken in the Scottish Learning Disabilities Observatory due to the growing concern among people with intellectual disabilities and their families around mortality. Its steering group includes people with intellectual disabilities, and partners from third sector organisations. Results from this study will be disseminated to people with intellectual disabilities and their families in an easy-read version via the Scottish Learning Disabilities Observatory website and newsletters.

For peer review only

For peer review only - http://bmjopen.bmj.com/site/about/guidelines.xhtml

Results

Out of 947,922 pupils in the census between 2008 and 2013 who were successfully linked to health records, there were 27,140 pupils who had ever registered as having an Additional Support Needs due to intellectual disabilities, and of these, 18,278 (1.9% of pupils) met the criteria of having at least two records of support. The remaining 8,862 pupils with a single support record were excluded, except for the sensitivity analysis. There were 909,688 pupils without any records of intellectual disabilities or autism. Of these, 131,776 were excluded due to appearing in only one year of the census, except for the sensitivity analysis. The remaining 777,912 pupils attended school for at least two years over the study period and were designated as controls.

Using data from the pupils' first year in the Census, pupils with intellectual disabilities were more likely to be male, and more likely to reside in areas of greater neighbourhood deprivation, and to have been registered for free school meals, compared to their peers (Table 1). Pupils with intellectual disabilities were also more likely to require adaptations in school, including physical adaptations, communication and curriculum adaptations. The majority of the study population were identified as having white (white -Scottish, -British or -other) ethnicity.

Missing education support records

There were 11,329 pupils (62%) of the intellectual disabilities group who appeared in certain census years without having a record of support. The majority, 70%, (n=7,970) were before the accrual of the first record; these pupils had a median 2 pupil census records prior to receiving their support (interquartile range (1,3)). There were 3,359 pupils or 18% of the entire study group who went on to have census records without support records, after having received intellectual disabilities support provision. These pupils had a median 1 subsequent year (IQR 1,2) without support, out of a median 4 remaining years (IQR 3,6) in the census.

For peer review only - http://bmjopen.bmj.com/site/about/guidelines.xhtml

Table 1. Demographic information for pupils with, and without intellectual disabilities

Demographic information [a]	Intellectual disabilities		Controls		p-value*
**Total, n (person-years)**	18,278	(67,342)	777,912	(3,672,224)	
**Male sex, n (%)**	11,891	(65%)	389,160	(50%)	p <0.001
**Age, person-years**					
<10	12,518	(19%)	995,297	(27%)	
10-14	28,297	(42%)	1,332,123	(36%)	
15-19	23,672	(35%)	1,178,608	(32%)	
19-24	2,855	(4%)	166,196	(5%)	
**Disability adaptations, n (%)**					
Physical adaptation, ever received	1,971	(11%)	1,837	(0.2%)	p <0.001
Curriculum adaptation, ever received	6,623	(36%)	6,341	(0.8%)	p <0.001
Communication adaptation, ever received	3,553	(19%)	1,760	(0.2%)	p <0.001
**SIMD quintile, n (%) at first census**					
1 (most deprived)	5,822	(32%)	169,038	(22%)	
2	3,888	(21%)	149,290	(19%)	
3	3,397	(19%)	152,415	(20%)	
4	2,896	(16%)	158,228	(20%)	
5 (least deprived)	2,275	(12%)	148,941	(19%)	p <0.001
**Ethnicity, n (%)**					
White [b]	16,553	(91%)	708,941	(91%)	p <0.001
Asian [b]	514	(3%)	23,791	(3%)	
Mixed or multiple ethnicities	144	(1%)	8,035	(1%)	
African, Caribbean or black	87	(<1%)	4,710	(<1%)	
Other ethnic groups	92	(<1%)	4,665	(<1%)	
Not disclosed / or unknown	888	(5%)	27,770	(4%)	

[a] Data taken from first census record, except for disability adaptation which includes any record across census years.
* χ^2-test for intellectual disabilities compared to control group (For SIMD - χ^2 test was across all categories, overall p value)
[b] (white -Scottish, -British, -Other) (Asian -Indian/British/Scottish, -Pakistani/British/Scottish, -Bangladeshi/British/Scottish, -Chinese/British/Scottish

For peer review only - http://bmjopen.bmj.com/site/about/guidelines.xhtml
For peer review only

Mortality analysis

Linking the pupil census population to the NRS registry of deaths up to February 2015 resulted in the equivalent of 3,739,568 person-years of follow up. There were 564 deaths identified in the study population during this period. There were 106 deaths (0.6%) among children and young people with intellectual disabilities over 67,342 person-years, which translated to a crude mortality rate of 157 deaths per 100,000 person-years (95% CI 130, 190). In the control group, there were 458 deaths (<0.1%) over 3,672,224 person-years, which translated to a crude mortality rate of 12 deaths per 100,000 person-years (95% CI 11, 14). The mean age of death among children and young people with intellectual disabilities was 14.3 years (95% CI 13.4 to 15.1) which was significantly lower (p<0.001) than controls where the mean age of death was 16.1 years (15.8 to 16.5). Sixty-two percent of deaths among children with intellectual disabilities occurred in males which was equivalent to the sex distribution in the whole intellectual disabilities cohort (p=0.545). Among controls, 61% of deaths occurred in males in spite of them accounting for only 50% of this group (p<0.001). Over 50% of deaths among pupils with intellectual disabilities occurred during childhood (<15 years old), compared to 29% of deaths among controls.

The all-cause age- and sex- SMR was 11.6 (95% CI 9.6, 14.0), as shown in Figure 1. The SMR was higher for female pupils than male pupils with intellectual disabilities; female SMR 16.6 (12.2, 22.6) versus male SMR 9.8 (7.7, 12.5). Exclusion of external causes of death resulted in a considerable increase in the all-cause SMR for both females and males with intellectual disabilities; overall SMR was 21.6 (17.8, 26.3), female SMR 25.6 (18.8, 34.9) versus male SMR 19.6 (15.3, 25.2). This produced a relative increase of 10 more deaths overall for pupils with versus without intellectual disabilities, which was similar in females (+9.0 increase), and males (+9.8 increase).The childhood (aged 5-14 years) SMR was 21.6 (16.6, 28.2) and was higher for females than males with intellectual disabilities; female SMR 30.3 (19.8, 46.5) versus male SMR 18.4 (13.1, 25.7). For young people (≥ 15 years old) SMR was 7.7 (5.9, 10.2) and was also higher for females than males with intellectual disabilities; female SMR 11.1 (7.1, 17.4) versus male SMR 6.5 (4.6, 9.3). Hence, the difference from the control pupils was greater in children rather than young people for both females and males.

The Cox-proportional hazards ratio for all-cause mortality, adjusted for age and sex, was found to be very similar; HR : 11.97 (9.64, 14.86). Proportional hazards assumption was met (p=0.4217). Kaplan-Meier survival curves for the overall time period are found in online supplementary data (Appendix 2).

For peer review only - http://bmjopen.bmj.com/site/about/guidelines.xhtml

- Insert Figure 1 about here -

Cause of Death

Cause of death data was available for over 95% of deaths among pupils with intellectual disabilities and over 91% deaths among controls. Table 2 shows the underlying causes of death and all-contributing causes of death by ICD 10 chapter. There were major differences between pupils with intellectual disabilities and controls with regards to the most common underlying causes. Among pupils with intellectual disabilities, these were diseases of the nervous system (33%), congenital malformations, deformations and chromosomal abnormalities (22%), followed by nutritional, metabolic and endocrine diseases (8%), of which most were conditions which were the cause of the pupils' intellectual disabilities e.g. neuronal ceroid lipofuscinosis or ornithine metabolism disorders. These were followed by respiratory diseases (8%) and neoplasms (7%). The most common underlying cause of death among control pupils was deaths due to external causes (46%), which made up a higher proportion of all deaths than in the pupils with intellectual disabilities (5%). Among controls, 71% of deaths due to external causes occurred in boys, compared with 100% in the intellectual disabilities group.

There were also differences in the most common all-contributing causes of death (Table 2). These chapters were not mutually exclusive, since one death could be included in several categories. Of the 106 deaths among pupils with intellectual disabilities, diseases of the nervous system contributed to 56 and diseases of the respiratory system contributed to 55. The 56 diseases of the nervous system included 34 due to cerebral palsy and 16 due to epilepsy. The 55 diseases of the respiratory system included 27 due to pneumonia, 9 due to pneumonitis associated with food and vomit, 17 due to respiratory failure, and 15 other respiratory disorders. In comparison, the control pupils had diseases of the nervous system contributing to 39 out of the total 458 deaths, and diseases of the respiratory system contributing to 51 of 458 deaths which included 21 due to pneumonia. The most common all-contributing causes of death for the control pupils were, as found for the underlying cause, external causes at 50% compared with 15% amongst pupils with intellectual disabilities.

Table 2 reports these data by presenting the cause-specific crude mortality rates by ICD 10 chapter for all pupils. As recommended by the ONS[19], avoidable mortality rates based on low numbers are labelled as unreliable and marked "U" or U.

For peer review only - http://bmjopen.bmj.com/site/about/guidelines.xhtml

Table 2. By ICD 10 chapter, the underlying causes of death as well as all-contributing factors in death, and cause-specific crude mortality rates per 100,000 person-years for pupils with, and without intellectual disabilities

ICD chapter[a]	Underlying cause of death								All-contributing factors in death							
	Intellectual disabilities				Controls				Intellectual disabilities				Controls			
	n (%)	CMR	95% CI		n (%)	CMR	95% CI		n	CMR	95% CI		n	CMR	95% CI	
Ch. 6. Diseases of the nervous system	35 (33%)	51.9	37.3	72.4	19 (4%)	0.5[U]	0.3	0.8	56	83.2	64.0	108.1	39	1.1	0.8	1.5
Ch. 17. Congenital malformations, deformations & chromosomal abnormalities	23 (22%)	34.2	22.7	51.4	13 (3%)	0.4[U]	0.2	0.6	32	47.5	33.6	67.2	18	0.5[U]	0.3	0.8
Ch. 4. Endocrine, nutritional & metabolic diseases	9 (8%)				15 (3%)	0.4[U]	0.2	0.7	11	16.3[U]	9.0	29.5	18	0.5[U]	0.3	0.8
Ch. 10. Diseases of the respiratory system	8 (8%)				17 (4%)	0.5[U]	0.3	0.7	55	81.7	62.7	106.4	51	1.4	1.1	1.8
Ch. 2. Neoplasms	7 (7%)				92 (20%)	2.5	2.0	3.1	8	Na			94	2.6	2.1	3.1
Ch. 20. External causes of morbidity & mortality	5 (5%)				210 (46%)	5.7	5.0	6.6	16	23.8[U]	14.6	38.8	231	6.3	5.5	7.2
Ch. 9. Diseases of the circulatory system	<5 (5%)				24 (5%)	0.7	0.4	1.0	15	22.3[U]	13.4	37.0	54	1.5	1.1	1.9
Ch. 11. Diseases of the digestive system	<5 (5%)				6 (1%)				13	19.3[U]	11.2	33.3	11	0.3[U]	0.2	0.5
Ch. 1. Certain Infectious & parasite diseases	<5 (5%)				12 (3%)	0.3	0.2	0.6	8				29	0.8	0.5	1.1
Ch. 5. Mental and behavioural disorders	<5 (5%)				<5 (1%)				6				17	0.5[U]	0.3	0.7
Ch. 3. Diseases of the blood, blood-forming organs & immune mechanism	<5 (5%)				<5 (1%)				<5				8			
Ch.14. Diseases of the genitourinary system	<5 (5%)				<5 (1%)				<5				<5			
Ch. 13. Diseases of the musculoskeletal system & connective tissue	0				<5 (1%)				<5				7			
Ch. 8. Diseases of the ear & mastoid process	<5 (5%)				0				<5				0			
Ch. 15. Pregnancy, childbirth & puerperium	0				<5 (1%)				0				<5			
Ch. 18. Symptoms, signs & abnormal clinical and laboratory findings[b]	Na				Na[b]				28				76			
Ch.19. Injury, poisoning & certain other consequences of external causes[b]	Na				Na[b]				10				219			
Unknown cause or error in underlying code	5 (5%)				39 (9%)				Na				Na			
**TOTAL**	106				458				Na				Na			

[a]n<5 repressed due to statistical disclosure CMR=crude mortality rate -reported for ≥10 deaths [U] Rates based on 10 - 20 deaths labelled "U" or unreliable

For peer review only - http://bmjopen.bmj.com/site/about/guidelines.xhtml

The top 10 individual leading causes of death are shown in table 3. Among pupils with intellectual disabilities, the highest number of individual underlying cause of deaths were cerebral palsy (18%), followed by congenital brain deformities (8%), and neoplasms (7%). Where there were fewer than five individual deaths per cause, these causes were not reported due to statistical disclosure control. For the majority of deaths in pupils with intellectual disabilities this was the case; 85% of specific causes could not be disclosed. Among control pupils, the highest number of individual underlying cause of deaths were neoplasms (20%), and road traffic accidents (17%). In relation to their peers, only three of the top ten underlying causes of death among children with intellectual disabilities featured in the top ten list for the controls – neoplasms (7% vs 20% of controls), epilepsy (5% vs 2% controls), and accidents (non-road traffic related, <5% vs 9% controls).

For peer review only

For peer review only - http://bmjopen.bmj.com/site/about/guidelines.xhtml

Table 3. The top ten specific underlying causes of death, and all-contributing causes of death, for pupils with, and without intellectual disabilities

Intellectual disabilities				Controls			
**Underlying cause of death**	**n**	**All-contributing factors**	**n**	**Underlying cause of death**	**n**	**All-contributing factors**	**n**
Cerebral palsy	19	Cerebral palsy	34	All neoplasms	92	Signs and symptoms: injury	114
Brain deformity	9	Pneumonia	27	Traffic accident	76	All neoplasms	94
All neoplasms	7	Respiratory failure	17	Self-harm	54	Traffic accidents	76
Muscular dystrophy	6	Epilepsy	16	Accidents, other	41	Self-harm	54
Epilepsy	5	Respiratory disorders	15	External, undetermined intent	25	Signs and symptoms: asphyxiation	51
Chromosomal abnormalities	5	Brain deformity	12	Asthma	14	Accident, other	43
Neuronal ceroid lipofuscinosis	<5	Chromosomal abnormalities	10	Assault	13	Signs and symptoms: poisoning	29
Pneumonia including influenza	<5	Pneumonitis due to food and vomit	9	Infections	12	All infections	29
Congenital heart disease	<5	All neoplasms	8	Epilepsy	8	External, undetermined intent	26
Accidents, other	<5	All infections	8	Cystic fibrosis	8	Pneumonia	21
Unknown causes	5	Ill-defined or ambiguous death	8	Unknown causes	39	Ill-defined or ambiguous death	58

For peer review only

For peer review only - http://bmjopen.bmj.com/site/about/guidelines.xhtml

Cause-specific SMRs, indirectly standardised using five-year age-bands and robust errors, are shown in Figure 2. For underlying causes, this was only possible for the two largest categories (by ICD 10 chapters); congenital malformations, deformations and chromosomal abnormalities, and diseases of the nervous system. For the all-contributing causes, the age-SMR for seven chapters were calculated. For congenital malformations, deformations and chromosomal abnormalities the SMR was 98.8[U] (69.9, 139.7), and for diseases of the nervous system, was 76.5 (58.9, 99.4). The ratios were also high for diseases of the digestive system at 63.7[U] (37.0, 109.7); and for diseases of the respiratory system at 55.3 (42.5, 72.1). Despite external causes contributing to a larger proportion of deaths among the control group, the mortality rate was still higher in the intellectual disabilities group than in the controls; the crude rate was 23.8[U] per 100,000 person-years, compared to 6.3[U] per 100,000 for the controls for external cause of death (either as the underlying cause or as a contributing factor). This produced an SMR of 3.6[U] (2.2, 5.8) demonstrating there is considerable over-representation in the intellectual disabilities group versus the controls.

- Insert Figure 2 about here -

Avoidable mortality

According to the UK ONS definition of avoidable mortality, (deaths which are amenable, preventable, or both), 19% of deaths in the intellectual disabilities cohort were classed as avoidable; 15% of deaths were amenable to treatment, and 6% were preventable. The majority of avoidable deaths (80%) were considered amenable to treatment, for their age group, including epilepsy, pneumonia and neoplasms. Among the control pupils, 63% of deaths were classed as avoidable, 16% were amenable to treatment, and 48% were preventable. The crude avoidable mortality rate for pupils with intellectual disabilities was higher, at 29.7[U] (19.2, 46.0) per 100,000 in pupils with intellectual disabilities, compared to 7.8 (7.0, 8.8) per 100,000 in the control pupils. The SMR was 3.6[U] (2.3, 5.5). Further breakdown of avoidable rates was possible for deaths that were amenable to healthcare; in the intellectual disabilities group, the amenable mortality rate was 23.8[U] (14.6, 38.8) per 100,000 versus 2.0[U] (1.6, 2.5) per 100,000 in controls; and the SMR was found to be 11.5[U], (7.0, 18.8).

Among pupils with intellectual disabilities, there were additional causes of death that the authors of this paper consider would have been amenable to health care: aspiration pneumonia; otitis media; megacolon; gastrointestinal haemorrhage; gastroenteritis; and contributing causes of death including gastro-oesophageal reflux, and urinary tract infections. These are not currently included within the ONS list of underlying causes.

For peer review only

For peer review only - http://bmjopen.bmj.com/site/about/guidelines.xhtml

Sensitivity Analysis

Of 27,140 pupils with at least one record of support due to intellectual disabilities, 65% were male, and compared to the main analysis group, there were significant reductions in frequency of school adaptations (physical disability reduced from 11% vs 9%, ($p<0.001$), curriculum adaptations from 36% to 31% ($p<0.001$), and communication adaptations from 19% to 16% ($p<0.001$). There were higher numbers of pupils in this group with years without intellectual disabilities support. There were 156 deaths in the intellectual disabilities group (134 per 100,000 person-years [114.2, 156.3]) compared to 684 deaths (13.8 per 100,000 [12.8, 14.8]) amongst the control group. The SMR for this sensitivity analysis was 9.5 (95% CI 8.1, 11.1), a change of minus 2 excessive deaths compared to the main analysis SMR. Mean age of death was similar in the sensitivity group, being 14.4 years (13.7, 15.1) in the intellectual disabilities group, and 16.2 (15.9, 16.5) in the control group. The ratio of deaths by sex were also very similar, with no difference for the intellectual disabilities group; 61% deaths were in males, similar to the proportion of males in the group ($p=0.306$), and an increase in male deaths amongst controls; 63% deaths were in males, whereas only 50% in the control group were male ($p<0.001$).

For peer review only

For peer review only - http://bmjopen.bmj.com/site/about/guidelines.xhtml

Discussion

Principle findings and interpretation

Our study is one of very few that has reported mortality rates among children and young people with intellectual disabilities, and is highly novel in reporting underlying, all-contributing, and the most common individual causes of death at this age, including cause-specific SMRs. We have demonstrated that children and young people in Scotland with intellectual disabilities have a 12-fold risk of death compared to their peers, rising to 22-fold on excluding external causes. Pupils with intellectual disabilities were also over-represented in deaths that were amenable to healthcare, and were approximately 3.6 times more likely to experience an avoidable death (albeit calculated using unreliably low rates). Children aged 5 -14 years with intellectual disabilities had a higher risk relative to peers (SMR 21.6), than the young people aged ≥15 years with intellectual disabilities (SMR 7.7). This difference reflects that, in the general population, there were considerably more deaths in young people than in children, especially for males, as opposed to more deaths of children than young people with intellectual disabilities. The SMR was higher for female pupils in both age groups, reflecting the higher death rate of males in the controls. Nervous system and respiratory causes of death were the most common among children and young people with intellectual disabilities, including deaths that would have been amenable to quality health care, such as epilepsy, pneumonia, and pneumonitis due to food and vomit. It is highly important to identify amenable deaths so that actions can be devised and taken. Causes of death among children and young people with intellectual disabilities were higher across several disease categories than for other children and young people, including diseases of the nervous system, digestive system, respiratory system, endocrine, nutrition and metabolic diseases, diseases of the circulatory system, and external causes.

Previous studies have demonstrated that there is an increased risk of sudden unexpected death in epilepsy among people with intellectual disabilities, however, in our study, the majority of deaths which listed epilepsy as a contributing factor, also listed pneumonia, so this does not appear to account for our findings.

Whilst external causes of deaths accounted for the greatest proportion of deaths among control children and young people (46%), especially in males, we found that external causes of death were still over-represented among children and young people with intellectual disabilities compared with their controls (partly due to inhalation of gastric contents, and inhalation of objects obstructing breathing). Trollor et al. (2017)[17] hypothesised that higher SMRs in adult women than men with intellectual disabilities may be driven by the larger proportion of male deaths in the general population due to external causes; and the lack of equivalent deaths in males with intellectual disabilities.

For peer review only - http://bmjopen.bmj.com/site/about/guidelines.xhtml

However, in our population of children and young people, when we re-calculated SMRs to exclude external causes, the observed increase in risk for females remained. Hence at this age range, this is only a partial explanation for the sex differences in SMRs, and there are further risk factors and vulnerabilities which require further exploration. It should be noted however, that in children and young people with intellectual disabilities, not all studies report a higher SMR in females compared with males[10, 11].

Comparison with previous studies

Two previous studies[9, 12] have reported a higher SMR for children than for young people. Glover et al. 2017[9] reported results separately for children aged 0-9 years, (SMR 30.4) and young people aged 10-17 years with intellectual disabilities (SMR 17.3). The Australian study by Bourke et al. 2017[12] reported a higher adjusted Hazard Ratio (aHR) for children aged 6-10 years (aHR 12.6) than young people aged 11-25 years (aHR 4.9). The SMRs we report are lower than those reported by Glover et al. (2017)[9], but the extent of difference between the children and young people is similar, albeit for differently defined age groups. The confidence intervals reported in our study are narrower due to the larger sample size. The SMRs we report are higher than those previously reported from small scale studies in Finland and USA[8, 10, 15], and a larger one in the Republic of Ireland[16], yet lower than a study reported from England[9], and a small study in Canada[11]. These differences may be due to actual international differences, or due to methodological differences between studies including: the method and source of identification of the population with intellectual disabilities; age ranges included; and study size with several of the previous studies having produced results with wide confidence intervals. All of these studies report a higher SMR in females than in males, except the study conducted in Canada and one in Finland.

The only previous study that has reported cause of death for children and young people aged 1-25 years, reported the most common causes of death to be infections in 50% (particularly respiratory infections in 34%), birth defects in 19% (particularly cardiac defect in 15%), and accidents in 11%, although by ICD 10 chapter deaths due to diseases of the respiratory tract were reported for 4.6%, infections and parasitic diseases were 3.1%, and external causes were 7.7%; and the most common were congenital malformations, deformations and chromosomal abnormalities in 29.1%, and diseases of the nervous system in 27.6%[12]. They did not report cause-specific SMRs by ICD 10 chapters, but crude numbers were proportionally higher for the children with intellectual disabilities for diseases of the nervous system, endocrine, nutritional and metabolic diseases, and congenital malformations, and lower for conditions originating in the perinatal period, external causes, or injury or poisoning[12]. We demonstrated diseases of the nervous system and respiratory system to be the most common causes

For peer review only - http://bmjopen.bmj.com/site/about/guidelines.xhtml

of death, and that cause-specific SMRs were raised across all of congenital malformations, deformations and chromosomal abnormalities, diseases of the nervous system, digestive system, respiratory system, endocrine, nutrition and metabolic diseases, circulatory system, and external causes.

Glover et al. (2017) graphed avoidable deaths in his study of children and adults[9]. We are unaware of any previous studies numerically quantifying amenable deaths among children and young people with intellectual disabilities.

Strengths and limitations

Our study drew upon data from an entire country, collected annually, and linked to national death records. It was large in scale, including over 18,000 children and young people with intellectual disabilities, and a large control population. A record of intellectual disabilities at school brings an entitlement to additional support so is likely to drive good recording in high income countries like Scotland. However, it only uses a binary definition for intellectual disabilities, therefore the study could not investigate mortality among people with different causes and severities of intellectual disabilities. Our study was not large enough to delineate cause-specific mortality ratios by sex, nor to study whether there are any ethnic variations. Use of death certificate data is known to have limitations[19], including inconsistent reporting and no reporting of severity of conditions. There may be some diagnostic overshadowing in death certificate data for people with intellectual disabilities, obscuring the events leading to death[21-23]. The ONS list of avoidable deaths does not include some that appear important among children and young people with intellectual disabilities, such as aspiration pneumonia, otitis media, megacolon, gastrointestinal haemorrhage, gastroenteritis, which featured as an underlying cause of death in our data. Additionally, death certificate data does not include wider determinants of health and death that may be implicated, such as being the target of discrimination or neglect.

Additionally, while we believe this population to be highly representative of children with intellectual disabilities across Scotland, we acknowledge that we were unable to access data on children not in school; there may be some under-ascertainment of children with intellectual disabilities with exceptional and complex health needs unable to attend school.

Conclusions and future directions

It is extremely important to study deaths among children and young people with intellectual disabilities, especially as so few studies have previously done so. Amongst the studies that have, there exists wide variation in the extent of reported inequality compared to other children and young

For peer review only - http://bmjopen.bmj.com/site/about/guidelines.xhtml

people, and wide confidence intervals, but all show a higher SMR. Our large study provides robust data that quantifies the extent of the difference; children and young people have a 12 times higher risk of death. A larger body of research exists for adults (rather than children and young people) with intellectual disabilities, and demonstrates substantial inequalities, and a high proportion of amenable deaths that could be addressed via reasonable adjustments in care provision. In our study, we have now reported that children and young people with intellectual disabilities also experience inequalities and experience amenable deaths. This is important, and we need a better understanding of it so that targeted improvements in care can start to be developed and delivered to reduce this inequality. Heslop et al (2014)[24] conducted a confidential inquiry into deaths of people with intellectual disabilities and made recommendations for improvements to practice regarding respiratory deaths, including aggressive monitoring and treatment of gastro-oesophageal reflux as well as postural and physical therapies. We have found that this is also important for children and young people with intellectual disabilities, if we are serious about improving life expectancy. Additionally, Scotland now offers influenza vaccines to all primary school aged children to reduce pneumonia; we therefore need to understand uptake by children with intellectual disabilities, and its determinants, to gauge whether this will change mortality findings.

The results of this study should be used to inform and direct multidisciplinary healthcare teams, as well as educators and carers to the associated risks of mortality in childhood and generate greater awareness around potential areas of improvement. Our countrywide study had a mean follow-up of around 5 years, and given that the pupil census is recorded annually, it presents the framework for further work to investigate both mortality trends in children and young people with intellectual disabilities, and a more detailed understanding of these. Future studies could consider looking at predictors of death in children and young people to inform translation of findings into clinical benefit for people with intellectual disabilities.

Acknowledgements: We thank the ScotXed Pupil Census, the National Records of Scotland, and Information Services Division (ISD) of NHS Scotland for providing data and the eData Research and Innovation Service team at ISD Scotland for assisting with the data linkage and analysis stage of this project.

Contributors: GS analysed the data, interpreted findings and wrote the first draft of the manuscript. MF developed record linkage, analysed the data, interpreted findings and contributed to the manuscript. JP developed record linkage, interpreted findings and contributed to the manuscript. DK, AH & CM interpreted data and contributed to the manuscript, S-AC conceived the study, analysed and interpreted the data, and contributed to the manuscript. All authors approved the final version of the manuscript.

For peer review only - http://bmjopen.bmj.com/site/about/guidelines.xhtml

Funding: This study was funded by an MRC Mental Health Data Pathfinder Award (MC_PC_17217), and the Scottish Government via the Scottish Learning Disabilities Observatory.

Disclaimer: The funders had no role in the study design, collection, analyses or interpretation of data, writing the report nor the decision to submit the article for publication.

Competing Interests: None declared.

Patient Consent: Not required.

Ethics approval: This study received approval from the NHS National Services Scotland Privacy Advisory Committee and Public Benefit and Privacy Panel -(PBPP) approval no. 1617-0259

Data sharing statement: This study linked patient information held across several administrative health datasets within ISD Scotland, with externally held data held by the Scottish Government (ScotXed education), and National Records of Scotland. Linkage and de-identification of data was performed by the Information Services Division (ISD) of NHS National Services Scotland (NSS). A data processing agreement between NHS NSS and University of Glasgow and a data sharing agreement between ScotXed and University of Glasgow was drafted. The University of Glasgow were authorised to receive record linked data controlled and held by ISD within NSS, via access through the national safe haven. The ISD Statistical Disclosure Control Protocol was followed. It is therefore not possible to share data with other parties.

For peer review only

For peer review only - http://bmjopen.bmj.com/site/about/guidelines.xhtml

References

1. Emerson E, Hatton C. Mental health of children and adolescents with intellectual disabilities in Britain. *Br J Psychiatry* 2007;191:493-499.
2. Hughes-McCormack LA, Rydzewska E, Henderson A, et al. Prevalence and general health status of people with intellectual disabilities in Scotland -a total population study. *J Epidemiol Community Health* 2017a;72:78–85.
3. Hughes-McCormack LA, Rydzewska E, Henderson A, et al. Prevalence of mental health conditions and relationship with general health in a whole country population of people with intellectual disabilities compared with the general population. *BJPsych Open* 2017b;3:243-248.
4. Heslop P, Lauer E, Hoghton M. Mortality in people with intellectual disabilities. *J Appl Res Intellect Disabil* 2015;28:367–372.
5. Hosking FJ, Carey IM, Shah SM, et al. Mortality among adults with intellectual disability in England: Comparisons with the general population. *Am J Public Health* 2016;106(8):1483–1490
6. O'Leary L, Cooper SA, Hughes-McCormack LA. Early death and causes of death of people with intellectual disabilities: A systematic review. *J Appl Res Intellect Disabil* 2018a;31:325–342.
7. O'Leary L, Hughes-McCormack LA, Dunn K, et al. Early death and causes of death of people with Down syndrome a systematic review. *J Appl Res Intellect Disabil* 2018b;31(5):687-708
8. Decouflé P, Autry A. Increased mortality in children and adolescents with developmental disabilities. *Paediatr Perinat Epidemiol* 2002;16(4):375–382
9. Glover G, Williams R, Heslop P, et al. Mortality in people with intellectual disabilities in England. *J Intellect Disabil Res* 2017;61(1):62–74
10. Patja K, Mölsä P, Iivanainen M. Cause-specific mortality of people with intellectual disability in a population-based, 35-year follow-up study. *J Intellect Disabil Res* 2001;45:30–40
11. Ouellette-Kuntz H, Shooshtari S, Balogh R, et al. Understanding information about mortality among people with intellectual and developmental disabilities in Canada. *J Appl Res Intellect Disabil* 2015;28(5):423–435
12. Bourke J, Nembhard WN, Wong K, et al. Twenty-Five Year Survival of Children with Intellectual Disability in Western Australia. *J Pediatr* 2017;188:232-239.e2.
13. Durvasula S, Beange H, Baker W. Mortality of people with intellectual disability in northern Sydney. *J Intellect Dev Disabil* 2002;27(4):255–264.
14. Scottish Index of Multiple Deprivation Background and Methodology, Scottish Government Website. https://www2.gov.scot/Topics/Statistics/SIMD/BackgroundMethodology (accessed July 2019)
15. Arvio M, Salokivi T, Tiitinen A, et al. Mortality in individuals with intellectual disabilities in Finland. *Brain Behav* 2016;6(2):1–4.
16. McCarron M, Carroll R, Kelly C, et al. Mortality rates in the general Irish population compared to those with an intellectual disability from 2003 to 2012. *J Appl Res Intellect Disabil* 2015;28(5):406–413
17. Trollor J, Srasuebkul P, Xu H, et al. Cause of death and potentially avoidable deaths in Australian adults with intellectual disability using retrospective linked data. *BMJ Open* 2017;7:e013489.
18. International Statistical Classification of Diseases and Related Health Problems 10th Revision, Volume 2 Instruction manual, 2010 edition. WHO. https://www.who.int/classifications/icd/ICD10Volume2_en_2010.pdf (accessed July 2019).
19. Office of National Statistics: Avoidable mortality in the UK Quality and Methodology Information. Office of National Statistics 2019. https://www.ons.gov.uk/peoplepopulationandcommunity/healthandsocialcare/causesofdeath/methodologies/avoidablemortalityinenglandandwalesqmi (accessed July 2019)

For peer review only - http://bmjopen.bmj.com/site/about/guidelines.xhtml

20. Olatunde O, Windsor-Shellard B, Campbell A. Revised definition of avoidable mortality and new definition for children and young people. Office for National Statistics 2016. https://www.ons.gov.uk/file?uri=/aboutus/whatwedo/statistics/consultationsandsurveys/allconsultationsandsurveys/reviewofavoidablemortalitydefinition/reviseddefinitionofavoidablemortalityandnewdefinitionforchildrenandyoungpeople.doc (accessed July 2019)
21. Landes SD, Peek CW. Death by mental retardation? The influence of ambiguity on death certificate coding error for adults with intellectual disability. *J Intellect Disabil Res* 2013;57(12):1183-1190
22. Glover, G, Ayub M. How people with learning disabilities die. Improving Health and Lives Learning Disability Observatory, Durham 2010. https://www.gov.uk/government/publications/people-with-learning-disabilities-in-england-2015 (accessed July 2019)
23. Landes SD, Stevens JD, Turk MA. Obscuring effect of coding developmental disability as the underlying cause of death on mortality trends for adults with developmental disability: a cross-sectional study using US Mortality Data from 2012 to 2016. *BMJ Open* 2019;9:e026614.
24. Heslop P, Blair PS, Fleming P, et al. The Confidential Inquiry into premature deaths of people with intellectual disabilities in the UK: a population UK: a population-based study. *Lancet* 2014;383(9920):889-895

For peer review only

For peer review only - http://bmjopen.bmj.com/site/about/guidelines.xhtml

Figure 1. Forest Plot of age-standardised and sex-specific mortality ratios for pupils with intellectual disabilities

Figure 2. Forest plot of cause-specific age-standardised mortality ratios for pupils with intellectual disabilities by ICD 10 chapter for underlying cause of death and for all-contributing factors of death

Footnote: Age-standardised mortality ratios (SMRs) & 95% confidence intervals were calculated using 5-year age bands for all ICD 10 chapters with ≥10 deaths. SMRs which were calculated using low numbers (between 10 and 20 deaths) are labelled "U" as unreliable

For peer review only - http://bmjopen.bmj.com/site/about/guidelines.xhtml

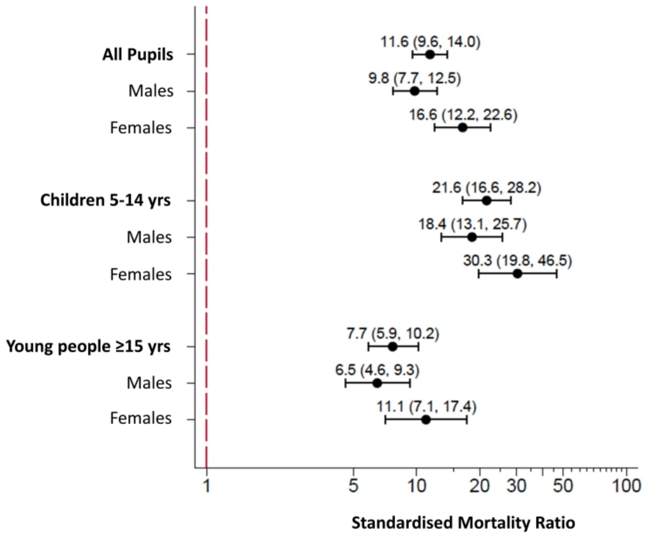

Figure 1. Forest Plot of age-standardised and sex-specific mortality ratios for pupils with intellectual disabilities

338x190mm (300 x 300 DPI)

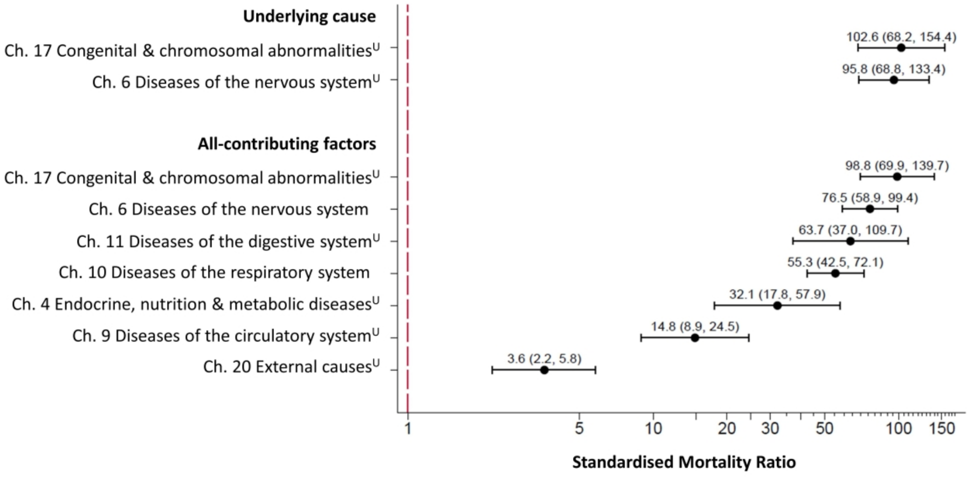

Figure 2. Forest plot of cause-specific age-standardised mortality ratios for pupils with intellectual disabilities by ICD 10 chapter for underlying cause of death and for all-contributing factors of death

338x190mm (300 x 300 DPI)

Supplementary – Appendix 1 - Reported standardised mortality ratios for children and young people with intellectual disabilities

**Author**	**Country**	**SMR overall (95% confidence interval) at all ages**	**Number of deaths of children/young people**	**SMR for children/young people**
McGuigan et al (1995)[1]	England	-	18 aged 0-19 y	1.7 (0.3, 4.9) for males aged 0-9 y*<br>3.1 (0.6, 9.0) for females aged 0-9 y*<br>17.1 (2.1, 61.8) for males aged 2-14 y*<br>20.7 (2.5, 74.7) for females aged 2-14 y*<br>22.2 (6.1, 56.8) for males aged 7-19 y*<br>34.0 (9.3, 87.1) for females aged 7-19 y*
Forsgren et al (1996)[2]	Sweden	2.0 (1.7, 2.3)	12 aged 0-19 y	15.5 (10.2, 23.6) aged 0-19 y
Patja et al (2001)[3]	Finland	-	50 aged 2-19 y; 29 profound ID, 5 Severe ID, 7 moderate ID, 8 mild ID	Death from disease: 2.6 for males, 1.7 for females aged 2-19 y<br>Accidental death: 0.1 for males, 0.1 for females aged 2 -19 y
Decouflé & Autry (2002)[4]	USA	_	23 aged 10-20 y	3.3 (2.1, 5.0) aged 10-19 y<br>1.4 (0.6, 2.9) with mild ID aged 10-19 y<br>8.4 (4.8, 13.6) with severe ID aged 10-19 y
Shavelle et al. (2014) [5]	USA	1.65 for mild and moderate ID<br>1.85 for severe and profound ID	59 aged 5-19 y mild and moderate ID<br>7 aged 5-19 y severe and profound ID	1.26** for mild and moderate ID aged 5-19 y<br>8.30** for severe and profound ID aged 5-19 y
Ouellette-Kuntz et al (2015)[6]	Canada	2.5 (2.1, 2.9)	11 males aged 0-19 y<br>3 females aged 0-19 y	21.6 (10.8, 38.7) for males aged 0-19 y<br>18.1 (3.7, 53.0) for females aged 0-19 y
Arvio et al (2016)[7]	Finland	2.3 (2.2, 2.4) for mild ID; 2.0 (1.9, 2.1) for males, 2.8 (2.6, 3.0) for females<br>3.4 (3.3, 3.5) for severe ID; 2.6 (2.5, 2.7) for males, 5.2 (5.0, 5.5) for females	8 with mild ID aged 0-14 y;<br>4 males, 4 females<br>110 with severe ID aged 0-14 y; 42 males, 68 female	4.2 (1.8, 8.3) with mild ID aged 0-14 y;<br>3.2 (0.9, 8.1) for males, 6.3 (1.7, 16.2) for females<br>13.3 (10.9, -) with severe ID aged 0-14 y;<br>8.2 (5.9, 11.1) for males, 21.4 (16.6, 27.1) for females
McCarron et al (2015)[8]	Republic of Ireland	3.9 (3.7, 4.0)	272 aged 0-19 y	6.9 (5.9, 7.5) aged 0-19 y
Lauer et al (2015) [9]	USA	1.19	58 age 18-24 y	5.9** aged 18-24 y
Glover et al (2017)[10]	England	3.2 (2.9, 3.4)	33 aged 0-17 y	30.4 (18.3, 47.5) aged 0-9 y<br>17.3 (9.4, 29.0) aged 10-17 y
Trollor et al (2017) [11]	Australia	1.3 (1.2-1.5)	aged 20-24 y not provided	4.5** aged 20-24 y**
Bourke et al (2017)[12]	Australia	-	326 aged 1-27 y	aHR=6.0 (4.8, 7.6) aged 1-5 y<br>aHR=12.6 (9.0, 17.7) aged 6-10 y<br>aHR=4.9 (3.9, 6.1) aged 11-25 y

aHR=adjusted hazard ratio; ID=intellectual disabilities; SMR=standardised mortality ratio; y=years; *by individual birth cohorts **mortality ratio calculated from age-specific mortality rates

For peer review only

Supplementary References

1. McGuigan SM, Hollins S, Attard M. Age-specific standardized mortality rates in people with learning disability. *J Intellect Disabil Res* 1995;39(6):527-531
2. Forsgren L, Edvinsson SO, Nystrom L, et al. Influence of epilepsy on mortality in mental retardation: An epidemiologic study. *Epilepsia* 1996;37(10):956–963.
3. Patja K, Mölsä P, Iivanainen M. Cause-specific mortality of people with intellectual disability in a population-based, 35-year follow-up study. *J Intellect Disabil Res* 2001;45:30–40
4. Decouflé P, Autry A. Increased mortality in children and adolescents with developmental disabilities. *Paediatr Perinat Epidemiol* 2002;16(4):375–382
5. Shavelle RM, Sweeney LH, Brooks JC. Comparative Mortality of Persons With Intellectual Disability in California: An Update (2000-2010) *J Insur Med* 2014; 44(3):158-63
6. Ouellette-Kuntz H, Shooshtari S, Balogh R, et al. Understanding information about mortality among people with intellectual and developmental disabilities in Canada. *J Appl Res Intellect Disabil* 2015;28(5):423–435
7. Arvio M, Salokivi T, Tiitinen A, et al. Mortality in individuals with intellectual disabilities in Finland. *Brain Behav* 2016;6(2):1–4.
8. McCarron M, Carroll R, Kelly C, et al. Mortality rates in the general Irish population compared to those with an intellectual disability from 2003 to 2012. *J Appl Res Intellect Disabil* 2015;28(5):406–413
9. Lauer E, McCallion P. Mortality of People with Intellectual and Developmental Disabilities from Select US State Disability Service Systems and Medical Claims Data *J Appl Res Intellect Disabil* 2015;28;394-405
10. Glover G, Williams R, Heslop P, et al. Mortality in people with intellectual disabilities in England. *J Intellect Disabil Res* 2017;61(1):62–74
11. Trollor J, Srasuebkul P, Xu H, et al. Cause of death and potentially avoidable deaths in Australian adults with intellectual disability using retrospective linked data. *BMJ Open* 2017;7:e013489.
12. Bourke J, Nembhard WN, Wong K, et al. Twenty-Five Year Survival of Children with Intellectual Disability in Western Australia. *J Pediatr* 2017;188:232-239.e2.

For peer review only

Supplementary – Appendix 2. Kaplan-Meier survival estimates for pupils with and without intellectual disabilities

For peer review only

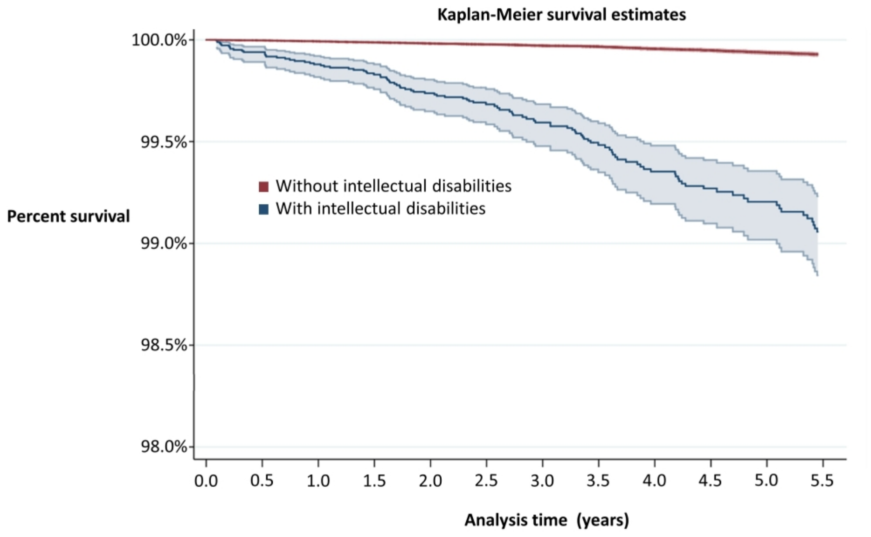

338x190mm (300 x 300 DPI)

STROBE Statement—Checklist of items that should be included in reports of ***cohort studies***

	Item No	Recommendation	Page No
**Title and abstract**	1	(*a*) Indicate the study's design with a commonly used term in the title or the abstract	1
		(*b*) Provide in the abstract an informative and balanced summary of what was done and what was found	1
**Introduction**			
Background/rationale	2	Explain the scientific background and rationale for the investigation being reported	3
Objectives	3	State specific objectives, including any prespecified hypotheses	4
**Methods**			
Study design	4	Present key elements of study design early in the paper	5
Setting	5	Describe the setting, locations, and relevant dates, including periods of recruitment, exposure, follow-up, and data collection	5
Participants	6	(*a*) Give the eligibility criteria, and the sources and methods of selection of participants. Describe methods of follow-up	5
		(*b*) For matched studies, give matching criteria and number of exposed and unexposed	na
Variables	7	Clearly define all outcomes, exposures, predictors, potential confounders, and effect modifiers. Give diagnostic criteria, if applicable	5
Data sources/ measurement	8*	For each variable of interest, give sources of data and details of methods of assessment (measurement). Describe comparability of assessment methods if there is more than one group	6
Bias	9	Describe any efforts to address potential sources of bias	7
Study size	10	Explain how the study size was arrived at	5
Quantitative variables	11	Explain how quantitative variables were handled in the analyses. If applicable, describe which groupings were chosen and why	6
Statistical methods	12	(*a*) Describe all statistical methods, including those used to control for confounding	6
		(*b*) Describe any methods used to examine subgroups and interactions	
		(*c*) Explain how missing data were addressed	
		(*d*) If applicable, explain how loss to follow-up was addressed	
		(*e*) Describe any sensitivity analyses	
**Results**			
Participants	13*	(a) Report numbers of individuals at each stage of study—eg numbers potentially eligible, examined for eligibility, confirmed eligible, included in the study, completing follow-up, and analysed	8
		(b) Give reasons for non-participation at each stage	
		(c) Consider use of a flow diagram	
Descriptive data	14*	(a) Give characteristics of study participants (eg demographic, clinical, social) and information on exposures and potential confounders	8
		(b) Indicate number of participants with missing data for each variable of interest	
		(c) Summarise follow-up time (eg, average and total amount)	
Outcome data	15*	Report numbers of outcome events or summary measures over time	10

For peer review only

For peer review only - http://bmjopen.bmj.com/site/about/guidelines.xhtml

Main results	16	(*a*) Give unadjusted estimates and, if applicable, confounder-adjusted estimates and their precision (eg, 95% confidence interval). Make clear which confounders were adjusted for and why they were included<br>(*b*) Report category boundaries when continuous variables were categorized<br>(*c*) If relevant, consider translating estimates of relative risk into absolute risk for a meaningful time period	10
Other analyses	17	Report other analyses done—eg analyses of subgroups and interactions, and sensitivity analyses	16
**Discussion**			
Key results	18	Summarise key results with reference to study objectives	17
Limitations	19	Discuss limitations of the study, taking into account sources of potential bias or imprecision. Discuss both direction and magnitude of any potential bias	19
Interpretation	20	Give a cautious overall interpretation of results considering objectives, limitations, multiplicity of analyses, results from similar studies, and other relevant evidence	20
Generalisability	21	Discuss the generalisability (external validity) of the study results	18
**Other information**			
Funding	22	Give the source of funding and the role of the funders for the present study and, if applicable, for the original study on which the present article is based	21

*Give information separately for exposed and unexposed groups.

Note: An Explanation and Elaboration article discusses each checklist item and gives methodological background and published examples of transparent reporting. The STROBE checklist is best used in conjunction with this article (freely available on the Web sites of PLoS Medicine at http://www.plosmedicine.org/, Annals of Internal Medicine at http://www.annals.org/, and Epidemiology at http://www.epidem.com/). Information on the STROBE Initiative is available at http://www.strobe-statement.org.

For peer review only

For peer review only - http://bmjopen.bmj.com/site/about/guidelines.xhtml
